# Molecular characterization of the PhiKo endolysin from *Thermus thermophilus* HB27 bacteriophage phiKo and its cryptic lytic peptide RAP-29

**DOI:** 10.3389/fmicb.2023.1303794

**Published:** 2024-01-19

**Authors:** Monika Szadkowska, Aleksandra Maria Kocot, Daria Sowik, Dariusz Wyrzykowski, Elzbieta Jankowska, Lukasz Pawel Kozlowski, Joanna Makowska, Magdalena Plotka

**Affiliations:** ^1^Laboratory of Extremophiles Biology, Department of Microbiology, University of Gdańsk, Gdańsk, Poland; ^2^Department of Biomedical Chemistry, Faculty of Chemistry, University of Gdańsk, Gdańsk, Poland; ^3^Department of General and Inorganic Chemistry, Faculty of Chemistry, University of Gdańsk, Gdańsk, Poland; ^4^Institute of Informatics, Faculty of Mathematics, Informatics and Mechanics, University of Warsaw, Warsaw, Poland

**Keywords:** bacteriophage, thermostable endolysin, antimicrobial peptide, lysin, extremophile

## Abstract

**Introduction:**

In the era of increasing bacterial resistance to antibiotics, new bactericidal substances are sought, and lysins derived from extremophilic organisms have the undoubted advantage of being stable under harsh environmental conditions. The PhiKo endolysin is derived from the phiKo bacteriophage infecting Gram-negative extremophilic bacterium *Thermus thermophilus* HB27. This enzyme shows similarity to two previously investigated thermostable type-2 amidases, the Ts2631 and Ph2119 from *Thermus scotoductus* bacteriophages, that revealed high lytic activity not only against thermophiles but also against Gram-negative mesophilic bacteria. Therefore, antibacterial potential of the PhiKo endolysin was investigated in the study presented here.

**Methods:**

Enzyme activity was assessed using turbidity reduction assays (TRAs) and antibacterial tests. Differential scanning calorimetry was applied to evaluate protein stability. The Collection of Anti-Microbial Peptides (CAMP) and Antimicrobial Peptide Calculator and Predictor (APD3) were used to predict regions with antimicrobial potential in the PhiKo primary sequence. The minimum inhibitory concentration (MIC) of the RAP-29 synthetic peptide was determined against Gram-positive and Gram-negative selected strains, and mechanism of action was investigated with use of membrane potential sensitive fluorescent dye 3,3′-Dipropylthiacarbocyanine iodide (DiSC_3_(5)).

**Results and discussion:**

The PhiKo endolysin is highly thermostable with melting temperature of 91.70°C. However, despite its lytic effect against such extremophiles as: *T. thermophilus*, *Thermus flavus*, *Thermus parvatiensis*, *Thermus scotoductus*, and *Deinococcus radiodurans*, PhiKo showed moderate antibacterial activity against mesophiles. Consequently, its protein sequence was searched for regions with potential antibacterial activity. A highly positively charged region was identified and synthetized (PhiKo_105-133_). The novel RAP-29 peptide lysed mesophilic strains of staphylococci and Gram-negative bacteria, reducing the number of cells by 3.7–7.1 log units and reaching the minimum inhibitory concentration values in the range of 2–31 μM. This peptide is unstructured in an aqueous solution but forms an α-helix in the presence of detergents. Moreover, it binds lipoteichoic acid and lipopolysaccharide, and causes depolarization of bacterial membranes. The RAP-29 peptide is a promising candidate for combating bacterial pathogens. The existence of this cryptic peptide testifies to a much wider panel of antimicrobial peptides than thought previously.

## 1 Introduction

An important goal of enzymes’ engineering is the enhancement of their thermal stability and activity at elevated temperatures (Huang et al., 2020). Often, the protein’s robustness to thermal challenges is a deciding factor for commercialization of a product and thermophiles are sources of many industrially relevant enzymes, such as thermostable α-amylases, lipases, proteases, antimicrobial enzymes and others (Henne et al., 2004).

Bacteriophages (phages for short) are viruses that infect bacteria (Clokie et al., 2011). Thermostable endolysins (enzymes produced by bacteriophages) are attracting increasing attention due to their potent antibacterial activity. Phages produce endolysins at the end of their lytic cycle to cleave specific bonds within peptidoglycan (PG) layer of the bacterial cell wall which together with internal turgor pressure causes the host cell’s lysis and release of the progeny virions to the environment (Haddad Kashani et al., 2018). This is called “lysis from within,” but since the first external usage of endolysins to eliminate *Streptococcus pyogenes* in infected mice (Nelson et al., 2001), endolysins are increasingly being used as potential therapeutic agents.

Endolysins are categorized by their catalytic specificity (Gerstmans et al., 2018). Glucosaminidases, muramidases, and lytic transglycosylases target bonds between N-acetylmuramic acid (NAM) and N-acetylglucosamine (NAG) in the glycan strands of PG. Endopeptidases cleave bonds within the peptide parts, and N-acetylmuramoyl-L-alanine amidases hydrolyze bonds between the lactyl group of NAM and the α-amino group of L-alanine of the stem peptide of PG of most bacterial species (Vollmer et al., 2008; Vermassen et al., 2019). Gram-positive bacteria do not have an outer membrane (OM), therefore endolysins can directly lyse PG from the outside. In Gram-negatives the OM forms a protective barrier that prevents large molecules (>600 Da), such as endolysins, to reach PG (Gutiérrez and Briers, 2020). However, studies concerning enzymes lysing Gram-negative pathogens are on the rise nowadays (Zampara et al., 2020; Islam et al., 2022; Son et al., 2023). Although rare, there are lysins with intrinsic antibacterial activity related to the presence of cryptic antimicrobial peptides (Lai et al., 2011; Lood et al., 2015; Plotka et al., 2019; Raz et al., 2019). For example, mesophilic *Acinetobacter baumannii* phage endolysin LysAB2 is composed of 185 amino acids (aa) with an N-terminal lysozyme-like domain and a C-terminal positively charged region (Lai et al., 2011). The C-terminal putative amphipathic helix of LysAB2 was proposed to bind the negatively-charged lipopolysaccharide (LPS) of the OM of Gram-negative bacteria, while the N-terminal catalytic domain cleaved specific bonds within PG, leading to bacterial cell lysis. As a proof of concept, a P0 peptide has been synthetized based on the LysAB2 C-terminal sequence (113–145 aa) and the minimum inhibitory concentration (MIC) of P0 against *A. baumannii* was determined to be 64 μM (Peng et al., 2017). However, the direct interaction of P0 with components of OM was not tested, as well as the activity of P0 against Gram-positive microorganisms was not determined (the parental endolysin LysAB2 has a broad Gram-negative and Gram-positive antibacterial activity).

Another example of a thermostable endolysin is Ts2631 enzyme from *Thermus scotoductus* bacteriophage vB_Tsc2631, active against *A. baumannii* and *Pseudomonas aeruginosa* at 37°C (Plotka et al., 2019). This lysin has intrinsic antibacterial activity based on a protruding N-terminal cationic part, but the exact mechanism of its antibacterial activity is yet to be elucidated (Plotka et al., 2019). The cationic N-terminal region is also characteristic for TSPphg endolysin from *Thermus* sp. TC4 phage TSP4 (Wang et al., 2020). The 20-residue N-terminal region of TSPphg (MRLPTKTSRFGYVHGQRNHE) containing six positively charged residues (underlined) was proposed to be responsible for exogenous enzyme activity against Gram-negative *Salmonella Paratyphi* B, *Escherichia coli* O157 and *Klebsiella pneumoniae*. Not all endolysins from thermophilic bacteriophages have positively charged regions and not all have a broad antibacterial spectrum (Żebrowska et al., 2022). For example, the TP84_28 endolysin from *Geobacillus stearothermophilus* phage TP-84 was only moderately active against *E. coli* DH11S strain (Żebrowska et al., 2022), while a thermophilic lysozyme from *Thermus aquaticus* phage φIN93 had lysed specifically only the *Thermus* spp. cells (Matsushita and Yanase, 2008).

Therefore, in this work we characterize an endolysin derived from *Thermus thermophilus* bacteriophage phiKo with similarity in the primary sequence to two thermostable endolysins previously discovered in our laboratory—Ts2631 and Ph2119 from *Thermus scotoductus* bacteriophages vB_Tsc2631 and MAT2119, respectively (Plotka et al., 2014, 2015). Molecular analysis of the PhiKo enzyme was performed and its antibacterial property against thermophilic and mesophilic bacteria was tested. Then, based on bioinformatics analysis, a peptide (RAP-29) with a potent antimicrobial function, hidden in the C-terminal region of this protein was found and synthesized (_105_RAPGWKSLAWLVRELRKHDSGLRLRLVRH_133_). Here, the bactericidal function and mechanism of action of this peptide were studied in detail.

## 2 Results

### 2.1 In search of lytic enzymes

**Figure 1:**
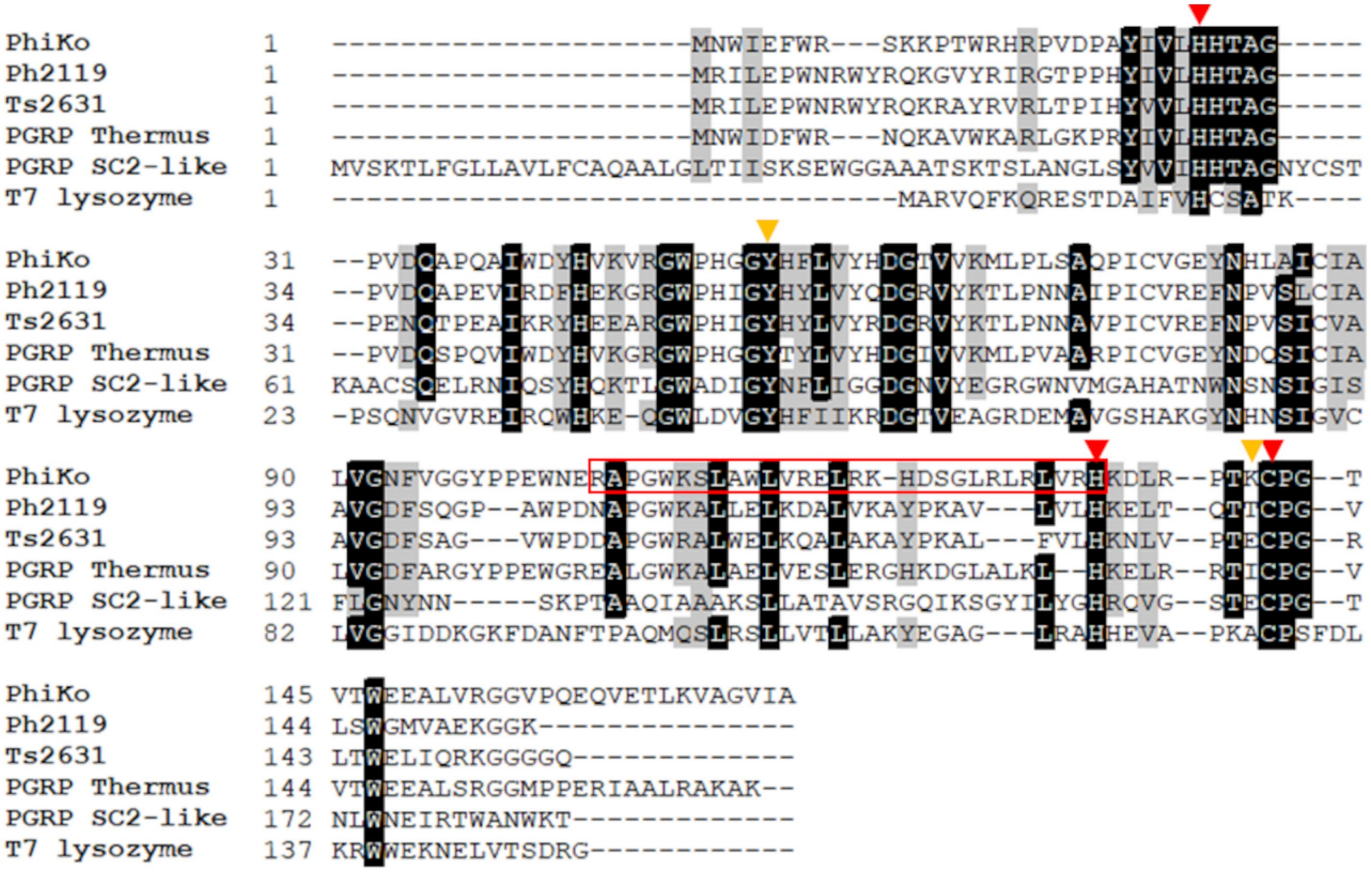
Multiple sequence alignment of PhiKo endolysin with similar proteins from the NCBI GeneBank database. Sequences of PhiKo endolysin (accession no AYJ74695.1), two thermostable endolysins Ph2119 and Ts2631 (accession no AHF20915.1 and AIM47292.1, respectively), PGRP from metagenome of *Thermus* sp. (accession no MDM7323587.1), PGRP SC2-like protein from *Musca domestica* (accession no NP_001295936.1), and T7 lysozyme (accession no AAB32819.1) were aligned with use of CLUSTAL Omega program. Black background represents 100% amino acid sequence identity, while gray shading reflects the amino acid conservation at 70% consensus. Red triangles indicate residues involved in Zn^2+^ binding: His27, His133 and Cys141. Yellow triangles highlight two additional residues, Tyr55 and Lys140 potentially responsible for PhiKo enzyme catalytic function. The sequence of RAP-29 peptide investigated within this work is marked with a red box.

The BlastN software was used to identify open reading frames (ORFs) encoding potential lytic proteins, similar to ORFs of two thermostable endolysins Ph2119 and Ts2631 active against Gram-negative bacteria, both derived from *T. scotoductus* phages, MAT2119 and vB_Tsc2631, respectively. This analysis has revealed in the unpublished genome sequence of *T. thermophilus* HB27 phage phiKo (accession no MH673671.2), an ORF (coordinates 2,728–3,243 nt) encoding a protein of 171 aa with a putative amidase activity. The calculated molecular mass of the protein is 19.5 kDa and an isoelectric point (pI) is 8.83. The identified enzyme (accession no AYJ74695.1) shows 67.1% primary sequence identity to PG recognition family protein (PGRP) from metagenome of *Thermus* sp. (accession no MDM7323587.1), 52.4% primary sequence identity to Ph2119 endolysin (accession no AHF20915.1), 49.0% identity to Ts2631 endolysin (accession no AIM47292.1), and 32.0% identity to T7 lysozyme (accession no ABN54767.1; Figure 1). According to Pfam classification, this protein (named by us the PhiKo endolysin) belongs to a family of N-acetylmuramoyl-L-alanine amidases (EC 3.5.1.28) and possesses a PF01510 domain (Amidase_2). That is not in line with the National Center for Biotechnology Information (NCBI) GenBank annotation of the PhiKo enzyme as a lysozyme and is most probably a result of PhiKo endolysin similarity to T7 lysozyme (Figure 1; Supplementary Figures 1, 2). The T7 lysozyme is an N-acetylmuramoyl-L-alanine amidase that hydrolyzes the amide bond between N-acetylmuramic acid (NAM) and L-Ala of the stem peptide of bacterial PG (Cheng et al., 1994). However, the generic term “T7 lysozyme” commonly found in the literature and public databases can be confusing (Schmelcher et al., 2012).

Results of Domain Enhanced Lookup Time Accelerated BLAST (DELTA-BLAST) show the PhiKo endolysin similarity to eukaryotic PG recognition proteins (PGRPs), e.g., PGRP SC2-like precursor from *Musca domestica* (29% primary sequence identity; Figure 1). According to the NCBI BLAST tool, the PhiKo endolysin resembles several uncharacterized N-acetylmuramoyl-L-alanine amidases encoded in the genomes of Terrabacterial group bacterium ANGP1 isolated from soil samples (Diamond et al., 2019), Microgenomates group bacterium Gr01-1014_7 isolated from groundwater well, and thermophilic *Anaerolineae* bacterium from volcano mud. The primary sequence identity between these proteins was between 31 to 39%, with query coverage of 71%–80%.

**Figure 2:**
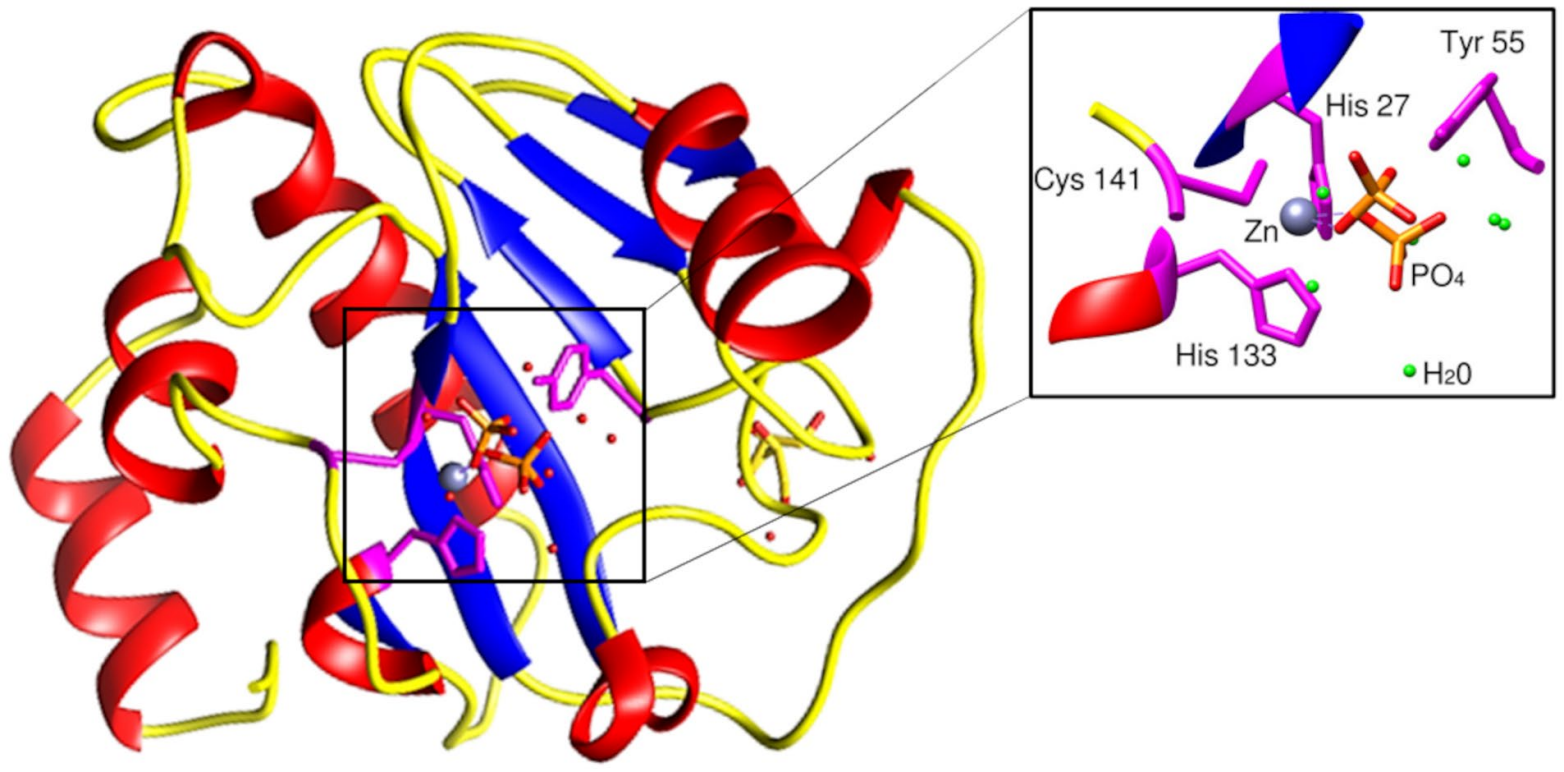
Structural model of the PhiKo endolysin. The secondary-structure elements: α-helices, β-strands, and loops are shown in red, blue, and yellow, respectively. His27, His133, Cys141, and Tyr55 [through a phosphate group (PO_4_^3−^)], represented as sticks, are involved in Zn^2+^ binding. Zn^2+^ ion is presented as a gray sphere (localization of the ion inferred by structural alignment to Ph2119 template, PDB:6SU5). Model had been predicted by ESMFold (Lin et al., 2023).

The BlastP results show conserved motifs in the primary sequence of PhiKo. Three residues, His27, His133, and Cys141 are predicted to form a Zn^2+^ binding site of the PhiKo endolysin that is a hallmark of type 2 N-acetylmuramoyl-L-alanine amidases (Figure 1). The Zn^2+^ coordinating residues and two other residues, Tyr55 and Lys140, are proposed to be responsible for the catalytic activity of this protein. A molecular model inferred by ESMFold (Lin et al., 2023) reveals that the overall architecture of the PhiKo endolysin consists of a five-stranded β-sheet that is flanked by six helices (Figure 2). The Zn^2+^ ion is placed in a cleft and is anchored to the protein by His27, His133, and Cys141, and through a phosphate group by Tyr55 (Figure 2; bioinformatics supplement available via RePOD; doi: https://doi.org/10.18150/8ZXKT8). However, Lys140, predicted to form the substrate binding site, is not directed toward the other residues in the catalytic center (Supplementary Figure 3A) and is not conserved in the primary sequence of the PhiKo endolysin (Figure 1).

Because PhiKo is the most similar to two thermostable endolysins, Ph2119 and Ts2631, active against Gram-negative bacteria, including *Pseudomonas aeruginosa* and *Acinetobacter baumannii*, both critical, priority pathogens according to WHO (Tacconelli et al., 2018), our natural choice was to experimentally explore the antibacterial potential of the PhiKo enzyme.

#### 2.1.1 Purification and oligomeric state of the enzyme

**Figure 3:**
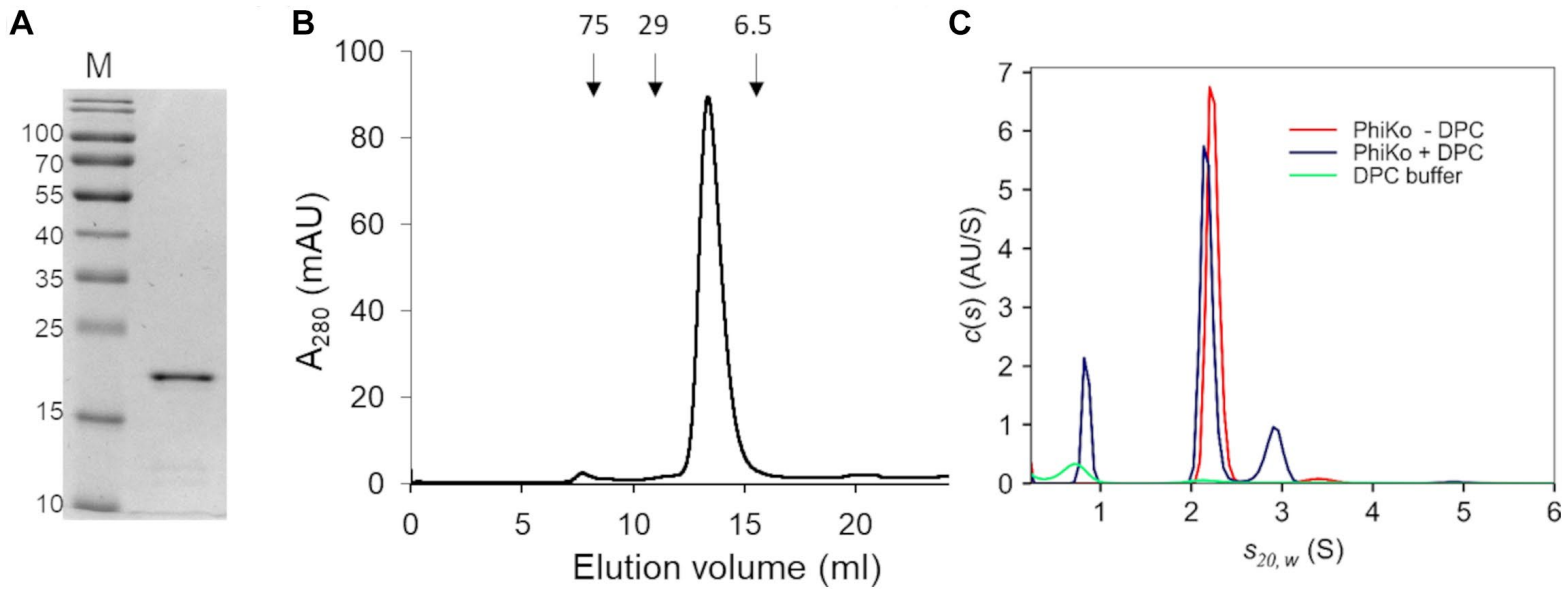
Purification and oligomeric state of the enzyme. **(A)** 12.5% SDS-PAGE gel of the purified PhiKo endolysin after dialysis into storage buffer (20 mM HEPES, pH 7.4, 50% glycerol). Predicted molecular mass of the recombinant enzyme with N-terminal His-tag is 21.7 kDa. M—marker proteins (PageRuler Prestained Protein Ladder, Thermo Scientific). The molecular masses of reference proteins (in kilodaltons) are indicated on the left. **(B)** Elution profile of PhiKo endolysin on Superdex 75 10/300 GL column. The position of molecular mass standards are shown as arrows, conalbumin 75 kDa, carbonic anhydrase 29 kDa, and aprotinin 6.5 kDa. mAU—milli-Absorbance Units **(C)** Degree of oligomerization of the PhiKo endolysin in the absence (red line) or presence (blue line) of n-dodecylphosphocholine (DPC) detergent. Green line corresponds to the control—buffer with DPC.

The recombinant PhiKo protein was purified by immobilized cobalt affinity chromatography and analyzed via SDS-PAGE electrophoresis. In the polyacrylamide gel, the protein migrated as a single band according to its predicted molecular mass of 21.7 kDa (Figure 3A). Size-exclusion chromatography was performed to analyze the oligomeric state of the PhiKo endolysin. This enzyme migrated as a single peak between carbonic anhydrase (29 kDa) and aprotinin (6.5 kDa), which indicated that this protein is highly pure, not prone to aggregation and migrates in a monomeric form (Figure 3B).

Analytical ultracentrifugation (AUC) was employed to analyze the degree of oligomerization of the PhiKo endolysin and to study the effect of the n-dodecylphosphocholine (DPC) detergent as an analog of bacterial cells’ membranes (Figure 3C). Detergents such as DPC have not previously been used to analyze the oligomerization of endolysins, but they have been successfully applied to induce the oligomerization of antimicrobial peptides interacting with bacterial membranes (Sancho-Vaello et al., 2020; Zeth and Sancho-Vaello, 2021). Therefore, samples of free protein and samples with DPC were centrifuged at 4°C in the course of the same experiment. In an independent, negative control (buffer with DPC, green line on the graph; Figure 3C) one peak was visible around 0.70 S_(20,w)_ which corresponds to sedimentation of DPC micelles. In samples with the PhiKo endolysin (red line) two peaks were present, the first one accounting for 97.7% of the signal and representing a monomer with a globular shape (frictional ratio 1.25). The second one (2.3% of the signal) indicated slight dimerization of the protein. By the nonlinear fittings, the average molecular mass of PhiKo endolysin was determined as 21.5 kDa which corresponds well with the molecular mass of 21.7 kDa calculated based on the recombinant protein sequence. In protein samples with DPC (blue lines), in addition to the peak corresponding to DPC micelles, similarly to the PhiKo endolysin alone, two peaks were present but the second one around 2.91 S_(20,w)_ corresponding to the dimer, was more prominent (15% of the total signal). The increased frictional ratio coefficient of 1.51 most likely explains the slightly slower sedimentation of this population. These results indicate protein tendency to form a dimer upon contact with bacterial cell membranes.

#### 2.1.2 Characterization of the PhiKo endolysin lytic activity

**Figure 4:**
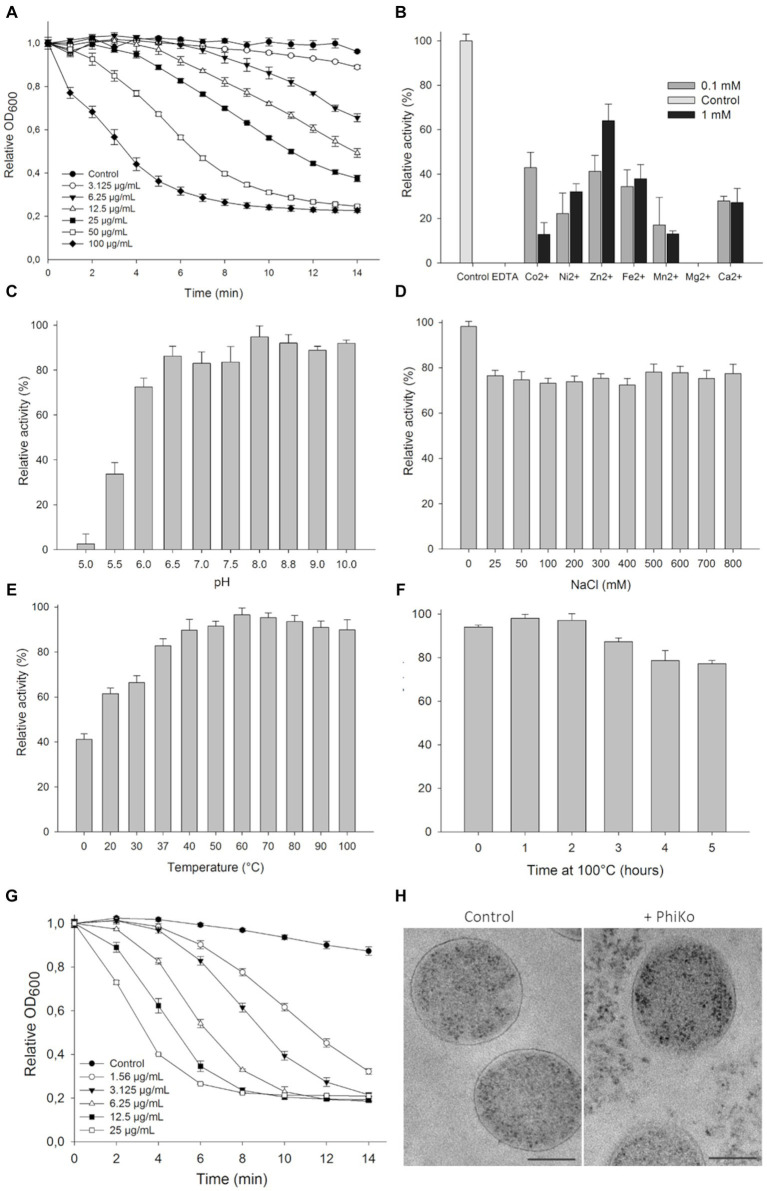
Lytic activity of PhiKo endolysin. **(A)** Dose dependent reduction in turbidity of chloroform-treated *Thermus thermophilus* HB8 cells suspended in 20 mM HEPES, pH 7.4. Increase in the enzyme activity was proportional to the enzyme concentration until saturation was achieved (100 μg/mL of endolysin within 10 min of assay). Error bars represent means ± standard deviations. Each experiment was repeated in triplicate. **(B)** Effect of divalent metal ions on PhiKo endolysin activity. The addition of 1 mM ZnCl_2_ restored the activity of the EDTA-treated enzyme to 63.1% compared to the control **(C)** Influence of pH on the PhiKo endolysin specific activity. Endolysin showed optimum activity at pH 8.0. **(D)** Effect of NaCl concentrations was assessed and maximum activity was achieved without salt addition and activity was above 70% at 25–800 mM of NaCl. **(E)** PhiKo endolysin withstands temperatures up to 100°C with above 80% activity at temp. Between 37°C and 100°C. **(F)** Activity of the PhiKo endolysin after heating at 100°C for 1 to 5 h in comparison to the untreated control. PhiKo residual activity was at the level of 77.3% ± 1.5% after 5 h heat-treatment. **(G)** Dose dependent reduction in *Thermus thermophilus* HB8 cell suspension turbidity under optimal conditions of 20 mM Tris–HCl, pH 8.0 at 60°C without salt addition. **(H)** Transmission electron microscope (TEM) observation of cell morphology of PhiKo-treated *T. thermophilus* HB8 cells. The scale bar represents 1 μm.

The lytic activity of the PhiKo endolysin was measured in a turbidity reduction assay against chloroform-treated *T. thermophilus* HB8 substrate in a universal 20 mM HEPES buffer, pH 7.4. The activity of the enzyme was dose dependent. With the increase of PhiKo concentration (3.125, 6.25, 12.5, 25, 50, and 100 μg/mL), the turbidity of *T. thermophilus* cells gradually decreased, reaching the lowest level at 100 μg/mL after 10 min of incubation (Figure 4A). The effect of divalent metal ions on the lytic activity of PhiKo endolysin was determined by turbidity reduction assay in the presence or absence of the following ions: Co^2+^, Ni^2+^, Zn^2+^, Fe^2+^, Mn^2+^, Mg^2+^, and Ca^2+^ (Figure 4B). No lytic activity of PhiKo was found upon treatment with EDTA, suggesting that this enzyme requires metal ions for efficient functionality. Except for Mg^2+^, all tested ions restored the activity of EDTA-treated PhiKo endolysin. The best effect was obtained after supplementation of the enzyme/bacteria suspension with 0.1 mM Co^2+^ and 1 mM Zn^2+^ (42.9 and 63.1% of the initial activity was achieved, respectively).

The PhiKo endolysin was most active between pH of 6.0–8.8, when the lysis exceeded 72.5% (Figure 4C). The activity was reduced to 33.6% at pH 5.5 and did not exceed 2.6% at pH 5.0. The PhiKo showed maximum lytic activity in the absence of NaCl but at salt concentrations of 25 to 800 mM this enzyme’s effectiveness remained at a similar level and did not drop below 72% (Figure 4D). The temperature optimal for this enzyme’s lytic activity was 60°C (Figure 4E). However, the enzyme was also active at 37°C (82.8% lysis) and in a lesser extent at 20°C (61.5% lysis). Moreover, the PhiKo enzyme showed more than 77% of residual activity after 5 h heating at 100°C (Figure 4F). Next, the activity of PhiKo endolysin was measured under optimal conditions, 20 mM Tris–HCl buffer, pH 8.0, without salt addition at 60°C. Again, the activity of the PhiKo endolysin was dose dependent, but already a concentration of 25 μg/mL resulted in a complete lysis of *T. thermophilus* HB8 substrate within 10 min (Figure 4G).

Transmission electron microscopy of *T. thermophilus* cells treated with 6.25 μg/mL of PhiKo endolysin for 10 min revealed PG layer degradation and outflow of cytoplasm outside the bacterial cell, contrary to the control where intact cell walls and well-defined PG layer were visible as a dark border (Figure 4H).

To verify experimentally the involvement of the Lys140 residue in the lytic activity of PhiKo endolysin, two variants Y55A and K140A were designed, overproduced, and purified, replacing Tyr55 and Lys140 by alanine. The Tyr55 residue was chosen for mutagenesis because it does not interact directly with the zinc ion in the catalytic center, but through the phosphate group (PO_4_^3−^). The activity of the variants compared to the wild-type protein was tested using the turbidity reduction assay. The results showed high activity of the K140A variant, comparable to the wild-type protein, while the Y55A variant was inactive. Therefore, the analyses show the involvement of the Tyr55 residue in the catalytic activity of the enzyme and the lack of such activity regarding Lys140 (Supplementary Figure 3B).

#### 2.1.3 Thermostability of the PhiKo endolysin

**Figure 5:**
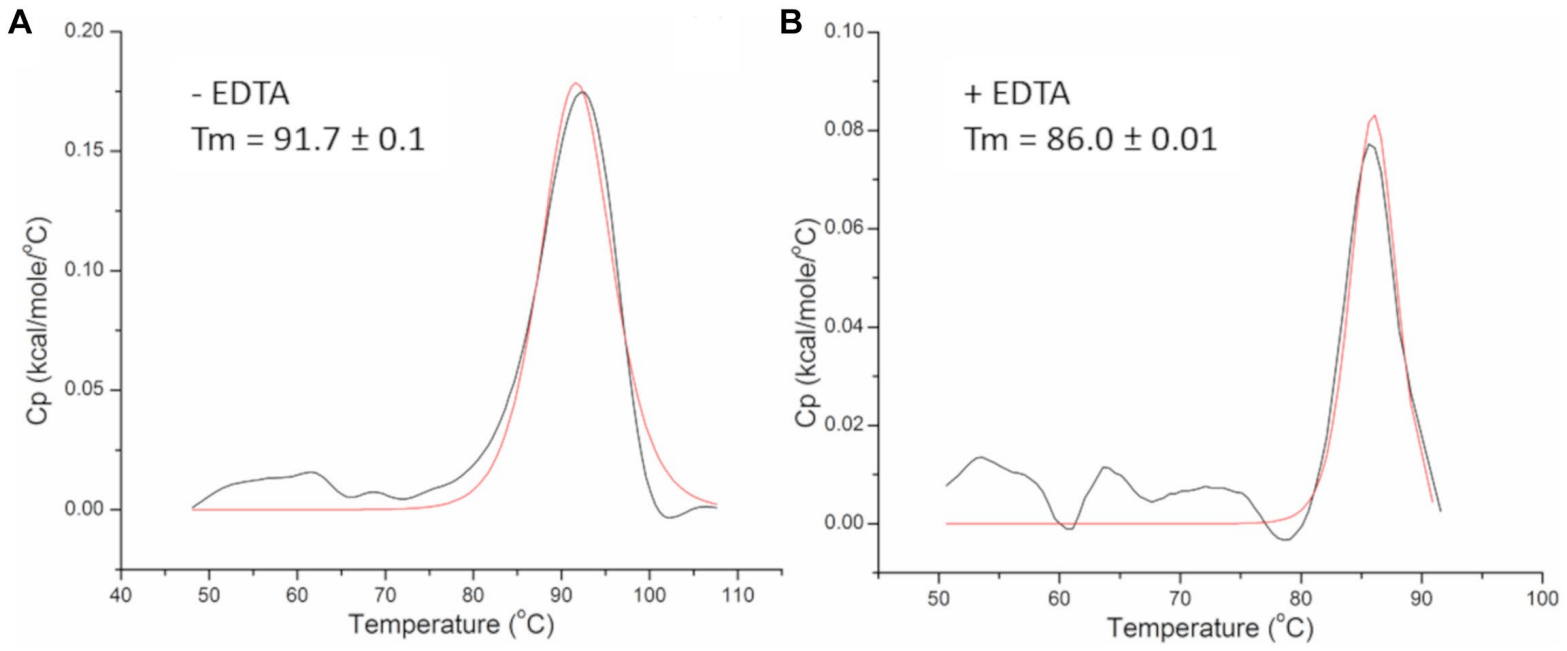
Heat-capacity curves of the PhiKo endolysin determined by DSC (black line) and the fit to the non-two state model (red solid line) in the absence **(A)** or presence **(B)** of 0.5 mM EDTA recorded in 20 mM MES, pH 6.0. Tm—melting temperature, the temperature at which 50% of the protein is unfolded.

Thermal stability of the PhiKo endolysin was assessed by differential scanning calorimetry (DSC; Figure 5). Analysis of the heat-capacity curve of the PhiKo endolysin revealed the heat-capacity peak at Tm = 91.7°C ± 0.1°C (Figure 5A). The transition point was estimated by fitting the experimental Cp vs. T curves to a non-two state model using Marquardt non-linear least squares method (Tm denotes the temperature value of the peak, the temperature at which a maximum of the endothermic effect emerges). Subsequent DSC experiments carried out with the addition of EDTA to the PhiKo endolysin showed that the newly formed system is less thermally stable (Tm = 86.0°C ± 0.01°C) when compared to the pure PhiKo, where the determined Tm was over five degrees higher (Figure 5B). This shows that the addition of EDTA reduces thermal stability of the PhiKo endolysin.

#### 2.1.4 Substrate specificity

**Figure 6:**
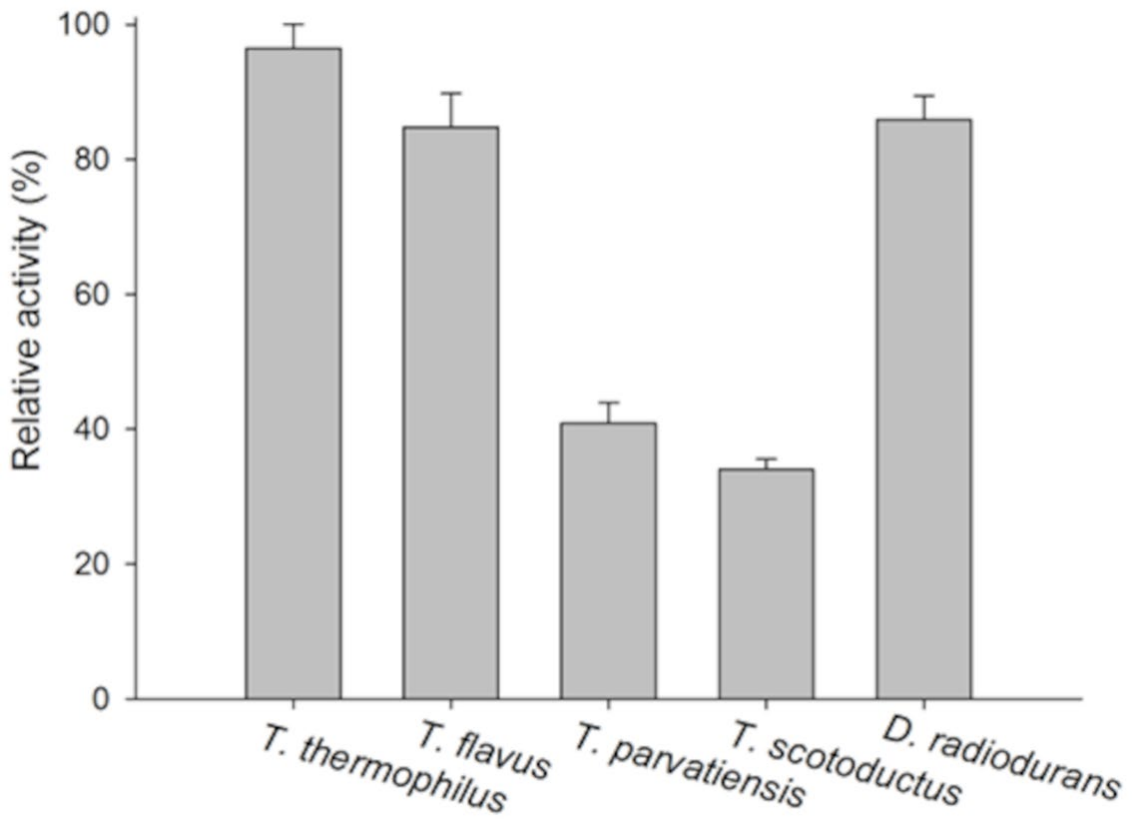
Substrate specificity of the PhiKo endolysin. Chloroform permeabilized Gram-negative bacteria *Thermus thermophilus* HB8, *T. thermophilus* HB27, *Thermus flavus* ATCC 1087, *Thermus parvatiensis* DSM 21745, *T. scotoductus* MAT2631, and Gram-positive *Deinococcus radiodurans* ATCC 13939 were prepared and used as a substrate in turbidity reduction assay. The 100% activity corresponds to the highest decrease obtained among the dataset. Experiments were performed in triplicate; error bars indicate standard deviations.

The lytic activity of the PhiKo endolysin was measured in a turbidity reduction assay against chloroform-treated substrates of extremophilic bacteria *Thermus thermophilus* HB8 DSM 579, *Thermus thermophilus* HB27 DSM 7039, *Thermus flavus* ATCC 1087, *Thermus parvatiensis* DSM 21745, *Thermus scotoductus* MAT2631 and *Deinococcus radiodurans* ATCC 13939 (Figure 6). The PhiKo endolysin lysed *T. thermophilus* HB8 the most effectively (average of 96.5% lysis), while *D. radiodurans* and *T. flavus* were lysed with slightly lower efficiency (85.9 and 84.8% lysis, respectively). *T. thermophilus* HB27 was lysed with an efficiency of 56.3%. The most resistant to the activity of the enzyme were *T. parvatiensis* (40.9%) and *T. scotoductus* (34.0%). Unfortunately, the PhiKo endolysin was not active against most substrates of mesophilic Gram-negative and Gram-positive bacteria, as shown by antibacterial tests (Supplementary Table 1). PhiKo endolysin showed activity exceeding 1 log CFU/mL reduction only against *Staphylococcus pettenkoferi* KPD 741 (4.40 ± 0.09 log killing) and *Acinetobacter baumannii* CRAB KPD 205 (2.57 ± 0.07 log killing). However, our attention was drawn to the possibility of PhiKo endolysin interaction with bacterial membranes, as indicated by the AUC results (Figure 3C).

### 2.2 Regions of PhiKo with antibacterial properties

**Figure 7:**
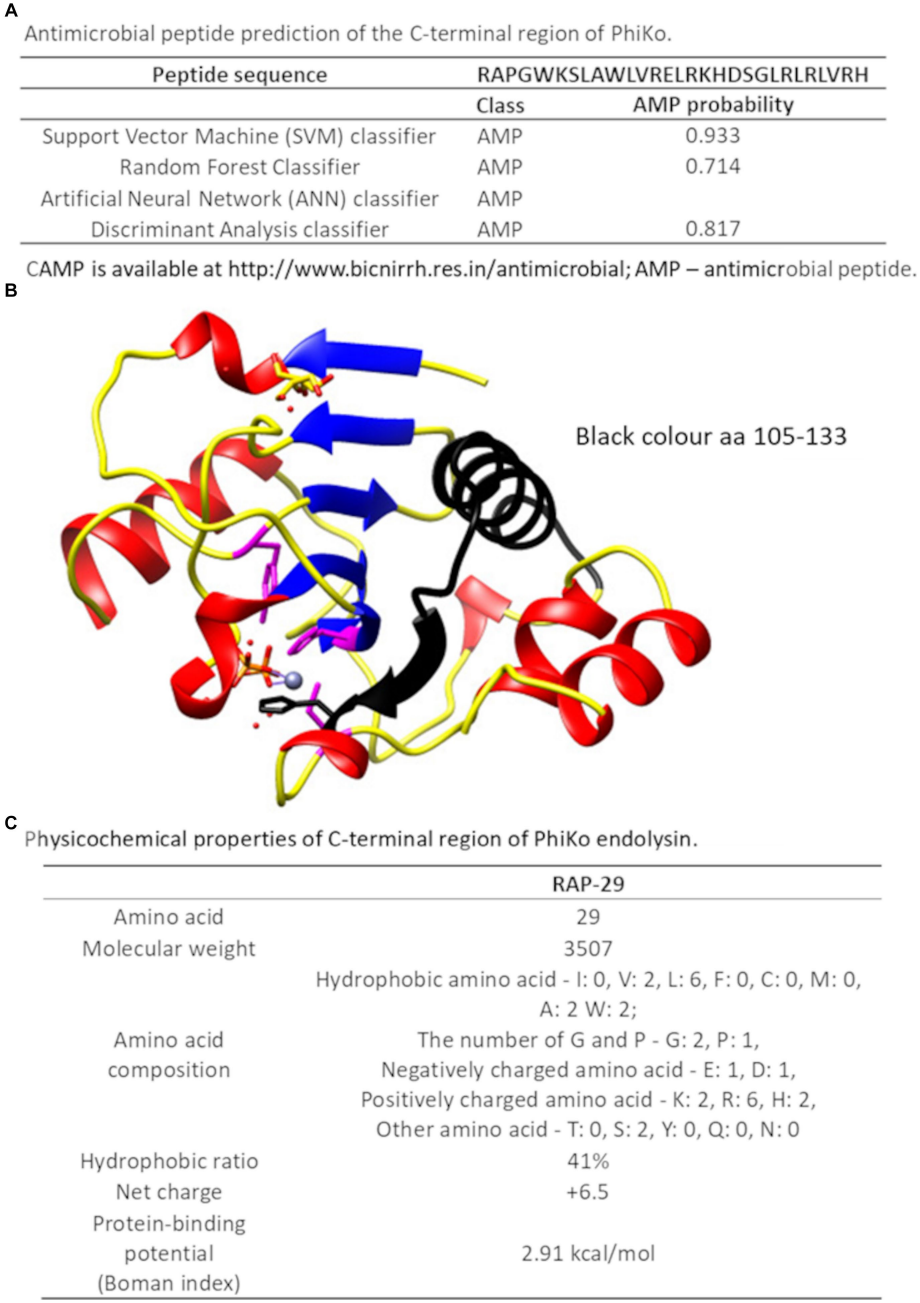
C-terminal region of the PhiKo endolysin with potential antibacterial properties **(A)** Antimicrobial peptide prediction of the C-terminal region of PhiKo endolysin with the use of Collection of Anti-Microbial Peptides (CAMP_R3_). A peptide is predicted to be antimicrobial if the probability is ≥0.5 **(B)** Structural model of PhiKo endolysin with the position of the peptide sequence highlighted in black **(C)** Physicochemical properties of the RAP-29 peptide analyzed by the Antimicrobial Peptide Calculator and Predictor (APD3).

Recently, in another lytic protein, LysC from *Clostridium intestinale* URNW, we found a region responsible for interaction with bacterial membranes and having antibacterial properties (Plotka et al., 2020; Szadkowska et al., 2022). Therefore, we employed the prediction tool of the Collection of Anti-Microbial Peptides (CAMP) and Antimicrobial Peptide Calculator and Predictor (APD3) in search of PhiKo protein regions with a potential antibacterial function. The CAMP is based on four different algorithms, three of which report results as numeric values from 0 to 1, and one algorithm designates protein regions as antimicrobial or non-antimicrobial. Few regions with potential activity against bacteria were found in the PhiKo sequence and the region PhiKo_105–133_, with the highest scores according to all algorithms, has been chosen for further analysis (Supplementary Table 2; Figure 7A). The PhiKo endolysin model shows that this region is formed by one α-helix and one β-sheet (Figure 7B). This peptide has been termed RAP-29, based on the first three residues (arginine R, alanine A, and proline P) and the length of 29 aa, has molecular weight of 3,507 Da, hydrophobic ratio of 41% and the net charge +6.5 (Figure 7C). Moreover, as predicted by APD3, RAP-29 may form an α-helix with eight residues on the same hydrophobic surface (underlined in the RAP-29 sequence _105_RAPGWKSLAWLVRELRKHDSGLRLRLVRH_133_). The most similar peptide in the APD3 database is HD5 (7–32) from *Homo sapiens* with an identity percentage of 38.24% (accession no AP03065) and activity against Gram-positive and Gram-negative bacteria (Ehmann et al., 2019).

#### 2.2.1 Antibacterial activity of RAP-29 peptide and MIC values

The antibacterial activity of RAP-29 was evaluated using standard antibacterial tests and selected Gram-positive and Gram-negative bacteria (Table 1). The RAP-29 peptide was synthetized based on the protein sequence, and then used to lyse 15 bacterial strains. The bactericidal activity was 3.7-log for *Pseudomonas aeruginosa* PAO1 and 7.1-log for multidrug-resistant *Acinetobacter baumannii* KPD 581. This peptide was also active against Gram-positive staphylococci exceeding 5.2-log activity against *Staphylococcus hominis* KPD 910.

**Table 1:** Bactericidal activity of the RAP-29 peptide against Gram-positive staphylococci and Gram-negative bacteria.

No.	Bacterial strain	Bactericidal activity [log reduction]
	**Gram-positive**	**RAP-29**
1.	*S. aureus* ATCC 25923	6.74 ± 0.06
2.	*S. aureus* KPD 740	6.69 ± 0.09
3.	*S. aureus* KPD 425	6.89 ± 0.03
4.	*S. epidermidis* KPD 440	6.93 ± 0.06
5.	*S. hominis* KPD 910	5.21 ± 0.06
6.	*S. pettenkoferi* KPD 741	6.47 ± 0.02
	**Gram-negative**	
7.	*A. baumannii* CRAB KPD 205	6.93 ± 0.06
8.	*A. baumannii* MDR KPD 581	7.10 ± 0.07
9.	*A. baumannii* KPD 735	6.95 ± 0.05
10.	*E. cloacae* KPD 297	3.78 ± 0.05
11.	*E. coli* KPD 217	6.17 ± 0.13
12.	*K. pneumoniae* KPD 298	4.41 ± 0.03
13.	*P. aeruginosa* KPD 430	6.65 ± 0.04
14.	*P. aeruginosa* CRPA KPD 431	5.72 ± 0.12
15.	*P. aeruginosa* PAO1	3.67 ± 0.10

Whereas the antibacterial tests were performed in a buffer, which alone may facilitate bacterial lysis due to high internal turgor pressure and low osmolality of the environment, the minimum inhibitory concentrations (MICs) were also determined and conducted in the Mueller Hinton broth. MICs were defined as the peptide concentration at which no bacterial growth was detectable after 24 h. The MICs of RAP-29 ranged from 2.0 to 31 μM, with the lowest MIC against *S. hominis* KPD 910 and the highest against two strains of *Staphylococcus aureus*, KPD 470 and KPD 425 (Table 2). The results of antibacterial tests, as well as the MIC values indicate that RAP-29 has antibacterial properties against mesophilic Gram-positive and Gram-negative bacteria.

**Table 2:** MICs of the RAP-29 peptide against Gram-positive staphylococci and Gram-negative bacteria.

No.	Bacterial strain	RAP-29 MICs	
	**Gram-positive**	**μg/mL**	**μM**
1.	*S. aureus* ATCC 25923	54.4	15.5
2.	*S. aureus* KPD 740	108.8	31.0
3.	*S. aureus* KPD 425	108.8	31.0
4.	*S. epidermidis* KPD 440	54.4	15.5
5.	*S. hominis* KPD 910	6.8	2.0
6.	*S. pettenkoferi* KPD 741	13.6	3.9
	**Gram-negative**		
7.	*A. baumannii* CRAB KPD 205	13.6	3.9
8.	*A. baumannii* MDR KPD 581	13.6	3.9
9.	*A. baumannii* KPD 735	13.6	3.9
10.	*E. cloacae* KPD 297	54.4	15.5
11.	*E. coli* KPD 217	27.2	7.8
12.	*K. pneumoniae* KPD 298	54.4	15.5
13.	*P. aeruginosa* KPD 430	54.4	15.5
14.	*P. aeruginosa* CRPA KPD 431	54.4	15.5
15.	*P. aeruginosa* PAO1	54.4	15.5

#### 2.2.2 Mechanistic insight into the function of RAP-29

**Figure 8:**
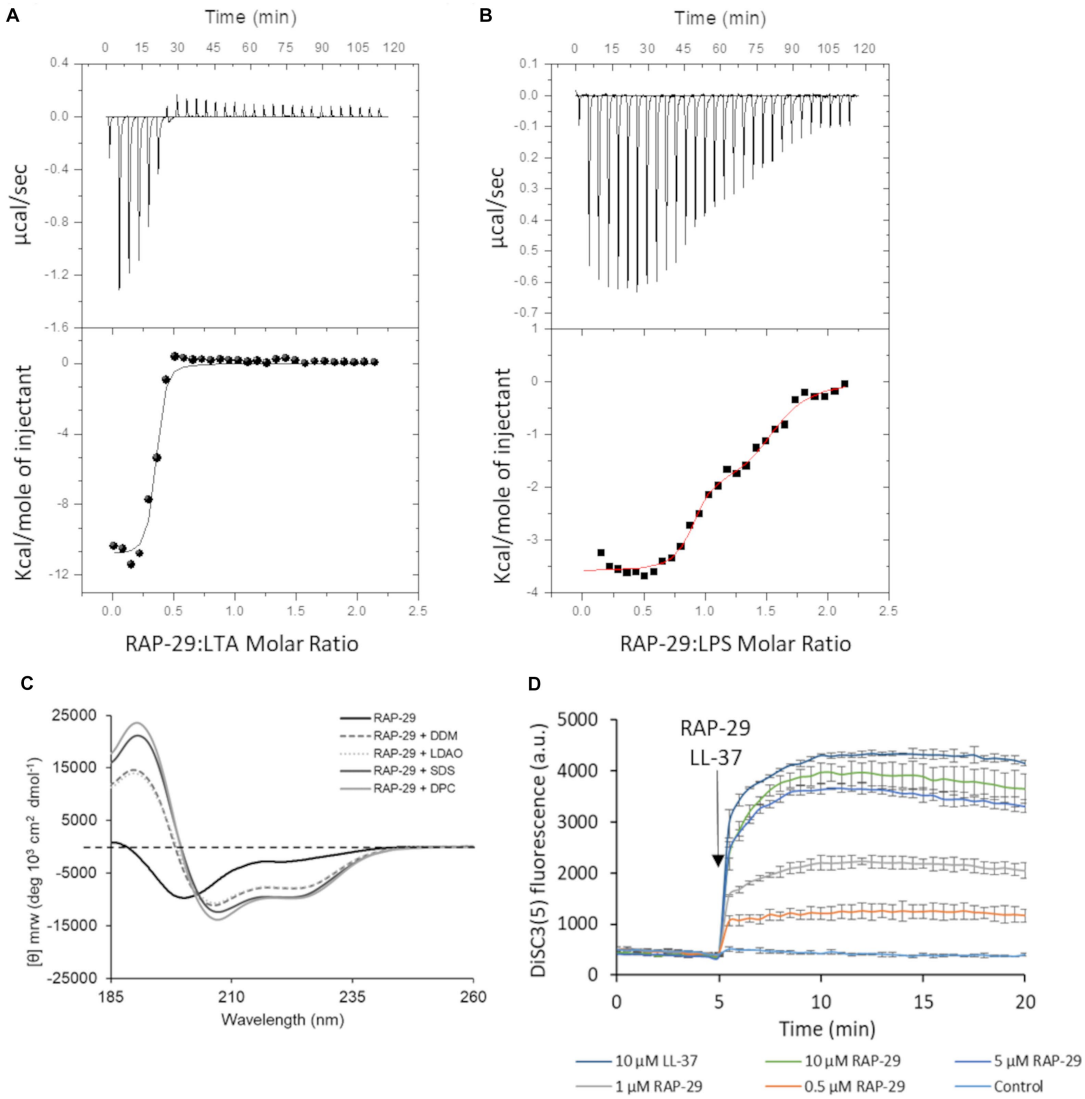
Mechanistic insights into the activity of RAP-29. Calorimetric titration isotherms of the binding interactions between RAP-29 and **(A)** lipoteichoic acid (LTA) of *Staphylococcus aureus* and **(B)** lipopolysaccharide (LPS) of *Pseudomonas aeruginosa* in 20 mM potassium phosphate buffer, pH 8.0 at 25°C. **(C)** Circular dichroism spectra of RAP-29 peptide show α-helical conformation of the peptide in the presence of 20 mM detergents DDM, LDAO, SDS, and DPC. Without addition of detergents RAP-29 is in a randomly coiled state; DDM—Lauryl-β-D-maltoside; LDAO—Lauryldimethylamine N-oxide; SDS—Sodium dodecyl sulfate; DPC—n-dodecylphosphocholine. **(D)** Bacterial membrane potential monitored with DiSC_3_(5) in the absence (control) or in the presence of 10 μM LL-37 (positive control) and increasing concentrations of RAP-29 (0.5 to 10 μM). The membrane potential sensitive dye DiSC_3_(5) was incubated with bacteria until a stable baseline was formed. The time point of RAP-29 and LL-37 addition is highlighted with an arrow. Fluorescence increase upon addition of peptides was measured at excitation wavelength of 652 nm and an emission wavelength of 672 nm as a function of time. Experiments were performed in triplicate; error bars indicate standard deviations.

To assess the possibility of the RAP-29 interaction with bacterial membrane components, isothermal titration calorimetry (ITC) measurements were performed with the use of lipoteichoic acid (LTA) isolated from Gram-positive *S. aureus*. The thermodynamic parameters of the RAP-29–LTA interaction, namely log*K*_ITC_ = 6.77 ± 0.16 and Δ*H*_ITC_ = −10.86 ± 0.26 kcal mol^−1^, were obtained directly from the ITC measurements by fitting isotherms (using nonlinear least-squares procedures) to a model that assumes one set of binding sites (Figure 8A). Then, the standard thermodynamic relationships: Δ*G*_ITC_ = −RTln*K*_ITC_ = Δ*H*_ITC_ − TΔ*S*_*I*TC_ were used to calculate the free energy of binding (Δ*G*_ITC_ = −9.24 ± 0.21 kcal mol^−1^), and the entropy change (TΔ*S*_ITC_ = −1.62 ± 0.33 kcal mol^−1^).

We found that RAP-29 forms a fairly stable complex with LTA (log*K*_ITC_ = 6.77 ± 0.16 M^−1^). Furthermore, under experimental conditions used (pH 8.0), formation of the RAP-29–LTA complex is an enthalpy-driven process (|Δ*H*_ITC_| > |TΔ*S*_ITC_|). The negative values of ΔH_ITC_ point to the fact that electrostatic interactions, mainly the charge–charge type between negatively charged hydrophilic chains of LTA and positively charged lysine, arginine and histidine of RAP-29, as well as van der Waals interactions and/or hydrogen bonds (Chakrabarti and Bhattacharyya, 2007; Panigrahi and Desiraju, 2007) involved in binding events, play a pivotal role in stabilization of the resulting RAP-29 – LTA complex. Due to the proven efficacy of the RAP-29 peptide against Gram-negative bacteria, we also assessed its affinity for the lipopolysaccharide (LPS) of *Pseudomonas aeruginosa* (Figure 8B). In contrast to LTA, two sets of binding sites capable of binding the RAP-29 peptide were detected for LPS. The stoichiometry of the resulting RAP-29–LPS complexes in both binding sites is close to 1:1. The thermodynamic parameters describing the investigated interactions are log*K*_ITC1_ = 7.78 ± 0.23 M^−1^, Δ*H*_ITC1_ = −3.59 ± 0.06 kcal mol^−1^, Δ*G*_ITC1_ = −10.61 ± 0.32 kcal mol^−1^, *T*Δ*S*_ITC1_ = 7.02 kcal mol^−1^ and log*K*_ITC2_ = 5.80 ± 0.17 M^−1^, Δ*H*_ITC2_ = −1.92 ± 0.02 kcal mol^−1^, Δ*G*_ITC2_ = −7.92 ± 0.23 kcal mol^−1^, *T*Δ*S*_ITC2_ = 6.00 kcal mol^−1^for the first and the second binding site, respectively. It is worth noticing that the binding of RAP-29 to LPS is favored by enthalpy and entropy changes, but unlike the RAP-29/LTA system, here the entropic factor prevails (|Δ*H*_(ITC)_| < |*T*Δ*S*_(ITC)_|).

Many antimicrobial peptides have a random coil structure in solution while in the presence of bacterial membranes they adopt an α-helical conformation (Sarkar et al., 2021). To verify this hypothesis, circular dichroism was performed for RAP-29 without or in the presence of micelles of Lauryl-β-D-maltoside (DDM), Dodecylphosphocholine (DPC), Lauryldimethylamine N-oxide (LDAO) and Sodium dodecyl sulfate (SDS). The RAP-29 peptide forms an α-helical structure in the presence of all tested microbial membrane mimics (Figure 8C) which suggests that this peptide acquires its secondary structure in the presence of a bacterial cell membrane. Next, the membrane potential was investigated upon addition of RAP-29 (Figure 8D). LL-37, the human cathelicidin peptide that dissipate the membrane potential, served as a positive control in these tests. The fluorescent carbocyanine dye DISC_3_(5) has been used to monitor bacterial membrane depolarization. This dye loses fluorescence intensity in polarized membranes and becomes highly fluorescent once polarization is lost. RAP-29 was able to rapidly depolarize the cytoplasmic membrane in a concentration-dependent manner. At the highest concentration of RAP-29 tested (10 μM), the level of depolarization appeared to reach a maximum at 5 min after peptide addition (Figure 8D). In summary, the RAP-29 peptide binds to the bacterial cell wall components, shows an α-helical structure in the presence of detergents DDM, DPC, LDAO, and SDS, and causes collapsing of the membrane potential which altogether leads to dysfunction of the bacterial cell.

## 3 Discussion

The versatile use of bacteriophages or their components, such as endolysins, e.g., in combating antibiotic-resistant bacteria or protecting food against bacterial contamination, most often refers to operating in a temperature range of 4°C–40°C (Liu et al., 2023). Advances in research on thermophilic bacteriophages and their proteins have opened new research avenues for their applications at elevated temperatures, e.g., in food processing conditions, including pasteurization.

A limited number of thermostable endolysins has been described in the literature, but their number grows constantly and this work fits well into this trend (Matsushita and Yanase, 2008; Ye and Zhang, 2008; Plotka et al., 2014, 2015; Wang et al., 2020; Choi and Kong, 2023; Jasilionis et al., 2023). Here, we characterize the PhiKo endolysin derived from extremophilic *T. thermophilus* HB27 bacteriophage phiKo. According to the NCBI BLAST, this protein possesses an amidase catalytic site with Zn^2+^ binding motif and cuts the amide bond between the glycan moiety (NAM) and the peptide moiety (L-alanine) of bacterial PG.

The group of thermostable endolysins includes, among others, LysGR1 endolysin from *Geobacillus stearothermophilus* phage GR1. This enzyme retains more than 60% of lytic activity after 15 min incubation at 70°C and lyses several foodborne pathogens, such as *Clostridium perfringens*, *Listeria monocytogenes*, and *Escherichia coli* O157:H7 (Choi and Kong, 2023). Another extremophilic enzyme, the Ts2631 endolysin of *Thermus scotoductus* phage vB_Tsc2631, caused 1.07–3.71 log_10_ reduction of *A. baumannii* and *P. aeruginosa* multidrug-resistant strains in antibacterial assays (Plotka et al., 2019). Subsequently, the MMPphg endolysin derived from thermophilic phage MMP17 was efficacious against Gram-positive and Gram-negative bacteria including nine antibiotic-resistant strains of *Klebsiella pneumoniae* (Wang et al., 2020). However, not all thermostable endolysins exhibit a broad spectrum of antimicrobial activity. The PhiKo endolysin, described herein, has a narrow spectrum. Similarly, the thermophilic lysozyme from bacteriophage φIN93 lyses specifically only the *Thermus* sp. cells (Matsushita and Yanase, 2008). The mechanism of such specificity is unknown and requires further studies. However, the *T. thermophilus* peptidoglycan represents a rare A3β chemotype with L-ornithine at position 3 of the stem peptide subunit (Schleifer and Kandler, 1972; Quintela et al., 1995), which may determine the high specificity of PhiKo endolysin of the *Thermus* bacteriophage. Gram-positive bacteria have mostly lysine (Lys) at position 3 of the stem peptide and Gram-negative bacteria mostly diaminopimelic acid (DAP; Schleifer and Kandler, 1972).

The PhiKo endolysin is thermally stable and its melting temperature is 91.70°C (Figure 5A). The Ts2631 endolysin that shows 49.0% primary sequence identity to PhiKo endolysin has T_m_ = 99.82°C (Plotka et al., 2015), and the Ph2119 endolysin (52.4% primary sequence identity with PhiKo) has T_m_ = 103.56°C (unpublished results). The melting temperature of another thermostable endolysin AmiP of *Thermus parvatiensis* prophage is 102.6°C (Jasilionis et al., 2023). As can be observed, the melting temperature of all mentioned endolysins differs only by a few degrees and is in a range of 91.70°C–103.56°C. For comparison, the newly discovered AbLys2 endolysin from metavirome of marine biofilm sample that shows lytic activity against mesophilic bacteria *A. baumannii* has a T_m_ of 47.1°C (Premetis et al., 2023). The addition of EDTA to PhiKo endolysin decreases the T_m_ to 86.0°C (Figure 5B). Since EDTA is a known metal ions chelator, and Zn^2+^ ion is present in the catalytic center of the PhiKo enzyme (Figure 2), its removal may result in destabilization of the enzyme structure or/and its inactivation.

The PhiKo endolysin displays its optimum lytic activity at pH 8.0 at 60°C without addition of NaCl. However, it retains more than 72% of initial activity in the presence of NaCl up to 800 mM. For example, *Pseudomonas aeruginosa* endolysins KZ144 from the giant phage φKZ tolerated high concentrations of NaCl and at a concentration of 500 mM the lytic activity of the enzyme was above 100% (Briers et al., 2007). On the other hand, increasing concentrations of NaCl inhibited the activity of LysB4 endolysin from *Bacillus cereus* phage B4 resulting in ~60% decrease in activity at 200 mM (Son et al., 2012). These results suggest that the effect of salt concentration on the enzyme activity is a unique property of a particular protein, and not a general trend related to endolysins. Under optimal conditions, PhiKo causes complete lysis of chloroform-treated *T. thermophilus* substrate within 10 min at a concentration of 25 μg/mL. Nevertheless, the narrow substrate specificity of the PhiKo endolysin precludes its usage as a potential antibacterial agent.

Recent research conducted in our laboratory on the thermostable Ts2631 endolysin revealed an N-terminal highly positively charged region in its amino acid sequence, most probably responsible for permeabilization of the outer membrane of Gram-negative bacteria (Plotka et al., 2019). A similar region with antibacterial properties was found in another lytic protein LysC derived from Gram-positive mesophilic bacterium *Clostridium intestinale* (Plotka et al., 2020; Szadkowska et al., 2022). Therefore, we decided to screen the amino acid sequence of PhiKo in search for similar regions. With use of the CAMP algorithm, several PhiKo regions with potential antibacterial activities were identified (Supplementary Table 2), and the region with the highest probability of antibacterial properties was selected for synthesis and further analysis.

The synthesized peptide, named RAP-29, is the first AMP derived from a thermophilic protein. In contrast to its parental enzyme PhiKo, RAP-29 showed strong antibacterial properties and lysed all Gram-positive and Gram-negative strains used in antibacterial assays with 3.7–7.1 log_10_ reduction of bacterial counts. Also, the MIC values ranged from 2 μM for *S. hominis* KPD 910 and 31 μM for *S. aureus* KPD 470 and *S. aureus* KPD 425. The obtained MICs for the RAP-29 peptide were significantly lower when compared to the MICs of another peptide derived from the lytic protein sequence, Intestinalin (P30) where the MIC values were often ≥124 μM (Szadkowska et al., 2022). Moreover, MICs values for peptide HD5 (7–32), which shares 38.24% primary sequence identity with RAP-29 were in a range of 25 μM (for *Acinetobacter baumannii* 4-MRGN and *Enterococcus faecium* 475747), and 50 μM for *Pseudomonas aeruginosa* ATCC 27853 (Ehmann et al., 2019).

The antimicrobial peptides are widespread in nature (Huan et al., 2020), and the cryptic peptides are AMPs that are embedded in larger proteins. An example of such a peptide is human lactoferricin which is released from the iron-binding glycoprotein lactoferrin through proteolysis by pepsin present in the gut. Lactoferricin consists of the N-terminal 49 residues of lactoferrin and similarly to RAP-29, possesses stronger antimicrobial activity than the parental enzyme (Gifford et al., 2005; Hunter et al., 2005). Human lactoferricin has the ability to bind LPS from the outer membrane of Gram-negative bacteria, and bovine lactoferricin (residues 17–41 of bovine lactoferrin) can bind teichoic acid originating from Gram-positive bacteria (Vorland et al., 1999). By the ITC analysis, similar properties of RAP-29 were revealed, as this peptide is able to bind both, the lipopolysaccharide and the lipoteichoic acid. Based on ITC data, it has been proven that LPS, in contrast to LTA, possesses two different sites capable of binding RAP-29. Moreover, the RAP-29 peptide causes bacterial membrane depolarization, as shown by fluorometric measurement with the use of membrane potential-sensitive dye DiSC_3_(5). Loss of membrane potential was also observed for lactoferricin and other antimicrobial peptides, such as gramicidin D (Boix-Lemonche et al., 2020; Gruden and Poklar, 2021).

Recently, cryptic peptides are gaining more attention. By using different algorithms, Nandi et al. (2022) predicted antimicrobial peptides from the protein sequences of *A. baumannii* specific phages. Anti-biofilm, anti-fungal, and cell-penetrating capacity of these peptides was predicted *in silico* and three peptides were selected as promising for experimental analysis (Nandi et al., 2022). In 2023, the American company ContraFect Corporation patented lysin polypeptides active against Gram-negative bacteria, such as *Pseudomonas aeruginosa*, *Klebsiella* spp., *Enterobacter* spp., *Escherichia coli*, *Citrobacter freundii*, *Salmonella typhimurium*, *Yersinia pestis*, and *Franciscella tulerensis* (Schuch et al., 2023). The peptide sequences were derived from five potential lysins GN37, GN2, GN4, GN14, and GN43 produced by phages. Studies have shown that in the presence of polymyxin B, the total pool of peptides named: PGN4, FGN4-1, FGN4-2, FGN4-3, and FGN4-4 (each at a concentration of 25 μg/mL) reduced the number of *P. aeruginosa* PAO1 cells in human serum by at least 2 log CFU/mL greater than the antibiotic alone.

To the best of our knowledge, our study is the first report on the presence of a peptide, hidden in the sequence of a larger thermophilic protein, with lytic properties against mesophilic Gram-positive and Gram-negative bacteria. These findings are especially important in the era of urgent search for new, effective antibacterial tools. As our results show, the cryptic antimicrobial peptide may have a broader antibacterial spectrum than the parental protein. That sheds new light on antibacterial peptides, indicating that the panel of effective antibacterial peptides may be broader than commonly thought. Therefore, studies of antibacterial peptides as an alternative strategy for antibiotics are still an inexhaustible and interesting direction of research in the light of growing drug resistance among pathogenic bacteria.

## 4 Materials and methods

### 4.1 Bacterial strains and culture conditions

Staphylococcal strains were cultured at 37°C in tryptic soy broth (TSB; Graso Biotech, Starogard Gdanski, Poland) or solid TSB medium containing 1.5% agar. All other mesophilic bacterial strains were grown at the same temperature in Luria-Bertani (LB) broth or LB agar. Thermophilic bacteria were grown in *Thermus* 162 medium [see the 878 position on the List of recommended media for microorganisms of the German Collection of Microorganisms and Cell Cultures GmbH (DSMZ)] at 60°C. All bacterial strains were received either from the American Type Culture Collection (ATCC), Collection of Plasmids and Microorganisms, University of Gdansk, Gdansk, Poland (KPD), DSMZ, or MATIS collection of microorganisms, Reykjavík, Iceland (MAT). Pattern of antibiotic resistance for each clinical strain is provided in Supplementary Table 3.

### 4.2 Bioinformatics analysis

A sequence-similarity search was performed using the Basic Local Alignment Search Tool (BLASTN for nucleotide and BLASTP for protein sequence) available at the National Center for Biotechnology Information web page.^1^ Protein sequences were aligned with CLUSTAL Omega software (Sievers et al., 2011). The isoelectric point (pI) and molecular weight of the PhiKo endolysin were determined using the IPC 2.0 tool (Kozlowski, 2021). The antibacterial properties of the C-terminal region of PhiKo endolysin and physicochemical properties of the RAP-29 peptide were predicted with the use of Antimicrobial Peptide Calculator and Predictor APD3 (Wang et al., 2016). The antimicrobial activity of the RAP-29 peptide was predicted by the use of Collection of Anti-Microbial Peptides CAMP which is based on four different algorithms: Support Vector Machine (SVM), Random Forest (RF), Artificial Neural Network (ANN), and Discriminant Analysis (DA; Thomas et al., 2010). The protein models were predicted by multiple methods, such as ESMFold (Lin et al., 2023), AlphaFold2 (Mirdita et al., 2022), OmegaFold (Sievers et al., 2011), RGN2 (Chowdhury et al., 2022), and RoseTTAFold (Baek et al., 2021) showing only minor differences (the critical residues were modeled consistently regardless of the method used). Furthermore, the models were processed, analyzed and visualized using USCF Chimera viewer (Pettersen et al., 2004).

### 4.3 DNA manipulations, overproduction and purification of PhiKo endolysin, and RAP-29 peptide synthesis

The gene coding for phage phiKo endolysin (accession no MH673671.2; locus_tag: phiKo_20; gene 2728.3243) was synthesized by BioCat GmbH, Heidelberg, Germany and was delivered as a plasmid DNA in a standard vector pET-15b carrying an N-terminal His-tag sequence. Prior synthesis, the nucleotide sequence of the *phiKo* gene was optimized by BioCat GmbH for efficient protein overproduction in *E. coli* BL21(DE3) strain (ThermoFisher Scientific). Site-directed mutagenesis was conducted with QuikChange II Site-Directed Mutagenesis Kit (Agilent). The primers used were Y55A_F (5′-GATAAACCAGAAAATGAGCACCACCATGAGGCCAACCAC GAA-3′), Y55A_R (5′-TTCGTGGTTGGCCTCATGGTGGTGCTCA TTTTCTGGTTTATC-3′) for Y55A variant, and K140A_F (5′-G TAACTGTACCCGGACATGCGGTCGGACGCAGATCTTT-3′), K140A_R 5′-AAAGATCTGCGTCCGACCGCATGTCCGGGTAC AGTTAC-3′ for K140A variant. The resulting recombinant plasmids were verified by DNA sequencing and transformed into *E. coli* BL21 (DE3) cells for protein overexpression.

Bacteria carrying the overexpression plasmid were cultivated at 37°C in 1 L of LB medium supplemented with 100 μg/mL ampicillin to an optical density at 600 nm (OD_600_) of 0.5. At that point, overproduction of the PhiKo endolysin or its substitution variants was induced by adding isopropyl-β-D-thiogalactopyranoside (IPTG) to a final concentration of 1 mM. Induction proceeded for 4 h at 37°C. Cells were harvested by centrifugation (10,000 × g for 20 min, 4°C) and resuspended in 20 mL of the NPi buffer (50 mM NaH_2_PO_4_, pH 8.0, 300 mM NaCl, 0.1% Triton X-100, 10% glycerol and 1 mM phenylmethylsulfonyl fluoride; PMSF) with 10 mM imidazole. The resuspended cells were sonicated (30 bursts of 10 s at an amplitude of 12 μm), followed by centrifugation and supernatant was loaded onto 1 mL HiTrap TALON crude column coupled to an HPLC chromatography system ÄKTA™ pure 25 (Cytiva). The protein was eluted with 150 mM imidazole in NPi buffer and dialyzed overnight against the HEPES buffer (20 mM HEPES, pH 7.4, 50% glycerol). The purity of the protein was assessed with the use of 12% sodium dodecyl sulfate-polyacrylamide gel electrophoresis (SDS-PAGE) and the concentration was determined by Bradford assay (Bradford, 1976). The protein was stored at −80°C until further analysis. The RAP-29 peptide synthesis was carried out exactly as previously described (Plotka et al., 2020). Size exclusion chromatography (SEC) analysis was performed using a Superdex 75 10/300 GL column on ÄKTA™ pure 25 chromatography system equilibrated with 25 mM NaH_2_PO_4_, pH 8.0, 150 mM NaCl. The column was loaded with 300 μL of PhiKo endolysin at a concentration of 1 mg/mL and the flow rate was set to 0.8 mL/min. The absorbance was measured at 280 nm (mAU, milli-absorbance units). Gel Filtration Calibration Kit Low Molecular Weight (Cytiva) was used to calibrate the column prior experiment.

### 4.4 Turbidity reduction assays

The peptidoglycan degrading activity of PhiKo enzyme was evaluated against outer membrane-permeabilized Gram-negative bacterial cells by measuring reductions in optical density at 600 nm (OD_600_). Substrates of *Thermus* strains were prepared using chloroform-Tris–HCl technique. Briefly, cells were grown at 60°C to an OD_600_ between 0.6–1.0, pelleted, and resuspended in 50 mM Tris–HCl, pH 7.7 saturated with chloroform. Samples were incubated for 45 min at room temperature with gentle shaking, washed with 50 mM Tris–HCl, pH 7.7, and lyophilized. Substrates were kept at −80°C, and before use were suspended in 20 mM HEPES, pH 7.4 (or indicated buffer) to an OD_600_ of 1.0. PhiKo endolysin (10 μL) was combined with chloroform-treated cells (190 μL) to achieve protein final concentration range of 1.56 to 100 μg/mL, and the change in OD_600_ was measured over time using EnSpire multimode plate reader (PerkinElmer). The lytic activity of PhiKo endolysin was calculated at specific condition as follows: (ΔOD_600_ control − ΔOD_600_ sample with endolysin)/initial OD_600_, as previously described (Plotka et al., 2015).

The pH dependence of PhiKo activity was assessed by incubating protein (final concentration 50 μg/mL) with 190 μL of *Thermus thermophilus* HB8 substrate resuspended in 20 mM sodium acetate (pH 5.0, 5.5, 6.0), 20 mM Tris–HCl (pH 6.5, 7.0, 7.5, 8.0, 8.8), 20 mM CAPS (pH 9.0), and 20 mM glycine (pH 10.0). The buffer with optimal pH (20 mM Tris–HCl, pH 8.0) was used to test the PhiKo endolysin activity in the presence of different concentrations of NaCl and temperatures in a range between 0°C and100°C. The PhiKo endolysin (final concentration 50 μg/mL) was heated at 100°C for 0-5 h and its residual activity was measured at 1 h time intervals.

To evaluate the effect of divalent metal ions on PhiKo endolysin function, *Thermus thermophilus* substrate was prepared as described above and suspended in 20 mM HEPES, pH 7.4 buffer with or without 0.1 mM and 1.0 mM ions (CoCl_2_, NiCl_2_, ZnCl_2_, FeCl_2_, Mn(CH_3_COO)_2_, MgCl_2_, and CaCl_2_). To chelate metal ions attached to the PhiKo endolysin, 5.0 mM EDTA was added to 5 μg of the enzyme and incubated for 30 min at 60°C. Subsequently, the endolysin was dialyzed overnight against 20 mM HEPES, pH 7.4. The lytic activity of EDTA-treated enzyme was compared to the one without EDTA treatment and without cation substitution. In all negative controls, 20 mM HEPES, pH 7.4 buffer instead of the PhiKo endolysin was added to the cell suspension. All experiments were performed in triplicate.

### 4.5 Antibacterial tests

The antibacterial activity of PhiKo endolysin was assayed against mesophilic bacteria listed in Supplementary Table 1. Briefly, bacterial cells were grown in TSB (staphylococci) or LB broth (other mesophilic bacteria) at 37°C to OD_600_ of 0.5. Bacterial cultures were harvested (4,000 × g for 15 min, 4°C), washed with 20 mM HEPES, pH 7.4 and diluted 100-fold in the same buffer to a final density of ~10^6^ cells/mL. Fifty μL of PhiKo endolysin or RAP-29 peptide were added to 400 μL of cells to achieve the final concentrations of 500 μg/mL and 20 μg/mL of the protein and peptide, respectively. After 90 min of incubation at 37°C, the mixtures were serially diluted and spread onto TSB or LB plates to determine the colony forming units (CFUs). The antibacterial activity was presented as the relative inactivation in logarithmic units (=log10 (N_0_/N_i_), where N_0_ = the number of residual cells (in the negative control) and N_i_ = the number of viable cells counted after incubation). For the negative control, the same volume of buffer was added instead of PhiKo endolysin or RAP-29 peptide. All experiments were performed in triplicate.

### 4.6 Minimum inhibitory concentrations

The MIC of the RAP-29 peptide was determined by the microdilution method in Difco™ Mueller-Hinton broth (MHB; ThermoFisher Scientific), as indicated by the Clinical and Laboratory Standards Institute (CLSI). Briefly, bacteria were grown overnight at 37°C and next day the number of mid-logarithmic bacterial cells was adjusted to 0.5 McFarland standard, diluted 100× to a density of 10^6^ CFU/mL and transferred into a 96-well round-bottom microtiter plate (90 μL/well). Two-fold serial dilutions of PhiKo peptide were prepared in MHB and placed in triplicate in a plate (10 μL/well) to achieve the final concentrations of the peptide ranging from 124 to 1.95 μM. The assays were incubated at 37°C for 24 h. MIC was determined as the lowest concentration of PhiKo peptide which completely prevented visible growth of the tested bacterial strain.

### 4.7 Transmission electron microscopy

Overnight culture of *T. thermophilus* HB8 cells was used to inoculate 20 mL of the 162 medium at 60°C and grown until exponential growth was reached. The cells were centrifuged, washed and resuspended in 20 mM Tris–HCl, pH 8.0 to a final concentration of ~10^7^ cells in a volume of 500 μL. The bacteria were incubated at 60°C for 10 min with PhiKo endolysin (6.25 μg/mL). As a control, buffer without protein was used. After incubation, bacteria were centrifuged and the pellet was fixed with 2.5% glutaraldehyde and post-fixed with 1% osmium tetroxide (Polysciences Inc.). Samples were dehydrated with ethanol and embedded in Epon 812 resin (Sigma-Aldrich). Ultrathin sections (60 nm) were obtained with ultramicrotome (Leica UC7), and stained with lead citrate and uranyl acetate (Sigma-Aldrich). TEM analyses were performed at the Laboratory of Electron Microscopy (Faculty of Biology, University of Gdansk, Poland). Bacteria were visualized using Tecnai Spirit BioTwin electron microscope (FEI Company).

### 4.8 Sedimentation velocity analytical ultracentrifugation

The SV-AUC experiments were performed using a ProteomeLab XL-I analytical ultracentrifuge (Beckman-Coulter, Inc.), equipped with An-50, 8-hole analytical rotor and double-sector charcoal-Epon cells (12 mm path length). The experiments were carried out at 50,000 rpm, in 20 mM MES, 150 mM NaCl, pH 6.0 in the presence or absence of 20 mM DPC (dodecylphosphocholine) detergent at 4°C, using continuous scan mode and radial spacing of 0.003 cm. 100 scans were collected with 8 min intervals between scans, both in the absorbance (280 nm) and interference mode. Cells were loaded with 400 μL of PhiKo endolysin (1.2 mg/mL) and 410 μL of buffer. Solvent densities (1.00851 g/cm^3^ without DPC and 1.00871 g/cm^3^ with DPC) and viscosities (1.581 without and 1.611 mPa s with DPC) were measured at 4°C using Anton Paar (Graz, Austria) DMA 5000 densitometer and Lovis 2000 M rolling-ball viscometer, respectively. Partial specific volume and extinction coefficients for proteins were calculated using the SEDNTERP software (Philo, 2011). Data were analyzed using the “Continuous c(s) distribution” model of the SEDFIT program (Schuck, 2000), with confidence level (F-ratio) specified to 0.68. The result were plotted using GUSSI graphical program (Brautigam, 2015).

### 4.9 Differential scanning calorimetry

For calorimetric experiments, the untreated or 5 mM EDTA-treated PhiKo endolysin was dialyzed against 20 mM MES, pH 6.0. The measurements were conducted on a VP-DSC microcalorimeter (MicroCal Inc.) with a scan rate of 90°C/h, a filtering period of 20 s, and with a prescan thermostat time of 15 min. To perform a DSC measurement of the protein unfolding process, the reference cell was filled with buffer, and the sample cell was filled with the PhiKo endolysin at a concentration of 1 mg/mL in 0.5 mL volume (before the mentioned step, the reproducibility of the baseline scan was done). PhiKo samples and buffers were degassed (5 min vacuum treatment) before loading into the calorimeter. All of the scans were run at a temperature range of 5°C to 110°C. The reversibility of the transition was checked by cooling and reheating the same sample and the measurements were recorded three times. The Origin 7.0 software for measuring instruments from MicroCal, was used for data analysis. The quantity measured by DSC is the difference between the heat capacity of MES buffer-protein solution and that of pure MES buffer. During DSC measurements, heat absorption causes a temperature difference (ΔT) between the cells when a protein unfolds.

### 4.10 Isothermal titration calorimetry

The ITC experiments were performed using the AutoITC isothermal titration calorimeter (MicroCal Inc.) at 25°C. The procedure was performed according to Panuszko et al. (2009). Briefly, lipoteichoic acid (LTA) from *S. aureus* or lipopolysaccharide (LPS) from *P. aeruginosa* 10 (both from SIGMA-Aldrich) and RAP-29 peptide were dissolved in 20 mM potassium phosphate buffer, pH 8.0, 10% glycerol. The RAP-29 peptide at concentration of 0.5 mM was gradually injected (29 injections for at 4-min intervals, 2 μL for the first injection only) into the reaction cell containing 0.05 mM solution of LTA or LPS. A background titration, consisting of the identical titrant solution but only the buffer solution in the sample cell, was subtracted from each experimental titration to account for heat of dilution. Mixing was carried out at constant 300 rpm. The standard procedure (CaCl_2_ − EDTA titration) was performed to validate the apparatus and the stoichiometry, *K*, Δ*H* results were compared with those obtained for the test kit from Malvern Instruments Ltd. (Malvern, United Kingdom).

### 4.11 Circular dichroism spectroscopy

To study the secondary structure of RAP-29 peptide, circular dichroism (CD) spectroscopy was performed with the use of Spectropolarimeter J-815 (Jasco) equipped with a 1-mm path length quartz cell. The spectra of RAP-29 (43 μM) were recorded at 25°C from 185 to 260 nm at 0.1-nm intervals. The peptide was dissolved in water or 20 mM micelles of Lauryl-β-D-maltoside (DDM), Dodecylphosphocholine (DPC), Lauryldimethylamine N-oxide (LDAO) and Sodium dodecyl sulfate (SDS). Each spectrum corresponded to the mean of six scans. The CD results were expressed as mean residue ellipticity (θ) in degree × cm^2^ × dmol^-1^.

### 4.12 Determination of membrane potential

Cells of mid-log phase *S. aureus* ATCC 25923, suspended at 1 × 10^6^ CFU/mL in 20 mM HEPES, pH 7.4, were incubated with 1 μM 3,3-Dipropylthiadicarbocyanine iodide dye DiSC_3_(5) in the presence of 1% DMSO. Cell suspension in a volume of 200 μL was added in triplicate to the wells of a black 96-well polystyrene microtiter plate (OptiPlate, PerkinElmer), and the plate was preincubated at 37°C for 25 min in the dark. Subsequently, fluorescence was monitored (excitation 652 nm, emission 672 nm) at 1 min intervals using EnSpire multimode plate reader (PerkinElmer). When the readings were stabilized, the plate was ejected, and RAP-29 peptide was added in triplicate to a final concentration between 0.5 μM to 10 μM. The fluorescence was measured again for 15 min at 1 min intervals. 10 μM LL-37 peptide (Sigma-Aldrich) served as a positive control.

### 4.13 Protein sequence accession numbers

The GenBank accession number for the protein sequence of the PhiKo endolysin is AYJ74695.

## Data availability statement

The raw files related to the bioinformatics analysis for this study are available under CC0 Creative Commons Zero 1.0 License and can be found in the RePOD repository (https://doi.org/10.18150/8ZXKT8).

## Author contributions

MS: Data curation, Investigation, Methodology, Writing – review & editing. AK: Visualization, Writing – original draft. DS: Investigation, Methodology, Writing – review & editing. DW: Data curation, Formal analysis, Investigation, Methodology, Visualization, Writing – review & editing. EJ: Formal analysis, Methodology, Supervision, Validation, Writing – review & editing. LK: Data curation, Methodology, Software, Validation, Visualization, Writing – review & editing. JM: Data curation, Formal analysis, Investigation, Methodology, Validation, Visualization, Writing – review & editing. MP: Conceptualization, Data curation, Formal analysis, Funding acquisition, Investigation, Methodology, Resources, Supervision, Validation, Visualization, Writing – original draft, Writing – review & editing.

## Footnotes

^1^https://blast.ncbi.nlm. nih.gov/blast.cgi

## References

[ref1] Baek M. DiMaio F. Anishchenko I. Dauparas J. Ovchinnikov S. Lee G. R.. (2021). Accurate prediction of protein structures and interactions using a three-track neural network. Science 373, 871–876. doi: 10.1126/science.abj8754, PMID: 34282049 PMC7612213

[ref2] Boix-Lemonche G. Lekka M. Skerlavaj B. (2020). A rapid fluorescence-based microplate assay to investigate the interaction of membrane active antimicrobial peptides with whole gram-positive Bacteria. Antibiotics 9:92. doi: 10.3390/antibiotics9020092, PMID: 32093104 PMC7168298

[ref3] Bradford M. M. (1976). A rapid and sensitive method for the quantitation of microgram quantities of protein utilizing the principle of protein-dye binding. Anal. Biochem. 72, 248–254. doi: 10.1016/0003-2697(76)90527-3, PMID: 942051

[ref4] Brautigam C. A. (2015). Calculations and publication-quality illustrations for analytical ultracentrifugation data. Methods Enzymol. 562, 109–133. doi: 10.1016/bs.mie.2015.05.001 26412649

[ref5] Briers Y. Volckaert G. Cornelissen A. Lagaert S. Michiels C. W. Hertveldt K.. (2007). Muralytic activity and modular structure of the endolysins of *Pseudomonas aeruginosa* bacteriophages phiKZ and EL. Mol. Microbiol. 65, 1334–1344. doi: 10.1111/j.1365-2958.2007.05870.x 17697255

[ref6] Chakrabarti P. Bhattacharyya R. (2007). Geometry of nonbonded interactions involving planar groups in proteins. Prog. Biophys. Mol. Biol. 95, 83–137. doi: 10.1016/j.pbiomolbio.2007.03.016, PMID: 17629549

[ref7] Cheng X. Zhang X. Pflugrath J. W. Studier F. W. (1994). The structure of bacteriophage T7 lysozyme, a zinc amidase and an inhibitor of T7 RNA polymerase. Proc. Natl. Acad. Sci. U. S. A. 91, 4034–4038. doi: 10.1073/pnas.91.9.4034, PMID: 8171031 PMC43717

[ref8] Choi D. Kong M. (2023). LysGR1, a novel thermostable endolysin from *Geobacillus stearothermophilus* bacteriophage GR1. Front. Microbiol. 14:1178748. doi: 10.3389/fmicb.2023.1178748, PMID: 37275144 PMC10237291

[ref9] Chowdhury R. Bouatta N. Biswas S. Floristean C. Kharkar A. Roy K.. (2022). Single-sequence protein structure prediction using a language model and deep learning. Nat. Biotechnol. 40, 1617–1623. doi: 10.1038/s41587-022-01432-w, PMID: 36192636 PMC10440047

[ref10] Clokie M. R. Millard A. D. Letarov A. V. Heaphy S. (2011). Phages in nature. Bacteriophage 1, 31–45. doi: 10.4161/bact.1.1.14942, PMID: 21687533 PMC3109452

[ref11] Diamond S. Andeer P. F. Li Z. Crits-Christoph A. Burstein D. Anantharaman K.. (2019). Mediterranean grassland soil C-N compound turnover is dependent on rainfall and depth, and is mediated by genomically divergent microorganisms. Nat. Microbiol. 4, 1356–1367. doi: 10.1038/s41564-019-0449-y, PMID: 31110364 PMC6784897

[ref12] Ehmann D. Wendler J. Koeninger L. Larsen I. S. Klag T. Berger J.. (2019). Paneth cell α-defensins HD-5 and HD-6 display differential degradation into active antimicrobial fragments. Proc. Natl. Acad. Sci. U. S. A. 116, 3746–3751. doi: 10.1073/pnas.1817376116, PMID: 30808760 PMC6397583

[ref13] Gerstmans H. Criel B. Briers Y. (2018). Synthetic biology of modular endolysins. Biotechnol. Adv. 36, 624–640. doi: 10.1016/j.biotechadv.2017.12.009, PMID: 29248682

[ref14] Gifford J. L. Hunter H. N. Vogel H. J. (2005). Lactoferricin: a lactoferrin-derived peptide with antimicrobial, antiviral, antitumor and immunological properties. Cell. Mol. Life Sci. 62, 2588–2598. doi: 10.1007/s00018-005-5373-z 16261252 PMC11139180

[ref15] Gruden Š. Poklar U. N. (2021). Diverse mechanisms of antimicrobial activities of Lactoferrins, Lactoferricins, and other Lactoferrin-derived peptides. Int. J. Mol. Sci. 22:11264. doi: 10.3390/ijms222011264, PMID: 34681923 PMC8541349

[ref16] Gutiérrez D. Briers Y. (2020). Lysins breaking down the walls of gram-negative bacteria, no longer a no-go. Curr. Opin. Biotechnol. 68, 15–22. doi: 10.1016/j.copbio.2020.08.014 33053478

[ref17] Haddad Kashani H. Schmelcher M. Sabzalipoor H. Seyed Hosseini E. Moniri R. (2018). Recombinant Endolysins as potential therapeutics against antibiotic-resistant *Staphylococcus aureus*: current status of research and novel delivery strategies. Clin. Microbiol. Rev. 31:17. doi: 10.1128/CMR.00071-17, PMID: 29187396 PMC5740972

[ref18] Henne A. Brüggemann H. Raasch C. Wiezer A. Hartsch T. Liesegang H.. (2004). The genome sequence of the extreme thermophile *Thermus thermophilus*. Nat. Biotechnol. 22, 547–553. doi: 10.1038/nbt956 15064768

[ref19] Huan Y. Kong Q. Mou H. Yi H. (2020). Antimicrobial peptides: classification, design, application and research Progress in multiple fields. Front. Microbiol. 11:582779. doi: 10.3389/fmicb.2020.582779, PMID: 33178164 PMC7596191

[ref20] Huang P. Chu S. K. S. Frizzo H. N. Connolly M. P. Caster R. W. Siegel J. B. (2020). Evaluating protein engineering Thermostability prediction tools using an independently generated dataset. ACS Omega 5, 6487–6493. doi: 10.1021/acsomega.9b04105, PMID: 32258884 PMC7114132

[ref21] Hunter H. N. Demcoe A. R. Jenssen H. Gutteberg T. J. Vogel H. J. (2005). Human lactoferricin is partially folded in aqueous solution and is better stabilized in a membrane mimetic solvent. Antimicrob. Agents Chemother. 49, 3387–3395. doi: 10.1128/AAC.49.8.3387-3395.2005, PMID: 16048952 PMC1196233

[ref22] Islam M. M. Kim D. Kim K. Park S. J. Akter S. Kim J.. (2022). Engineering of lysin by fusion of antimicrobial peptide (cecropin a) enhances its antibacterial properties against multidrug-resistant. Front. Microbiol. 13:988522. doi: 10.3389/fmicb.2022.988522, PMID: 36225352 PMC9549208

[ref23] Jasilionis A. Plotka M. Wang L. Dorawa S. Lange J. Watzlawick H.. (2023). AmiP from hyperthermophilic *Thermus parvatiensis* prophage is a thermoactive and ultrathermostable peptidoglycan lytic amidase. Protein Sci. 32:e4585. doi: 10.1002/pro.4585, PMID: 36721347 PMC9929850

[ref24] Kozlowski L. P. (2021). IPC 2.0: prediction of isoelectric point and pKa dissociation constants. Nucleic Acids Res. 49, W285–W292. doi: 10.1093/nar/gkab295, PMID: 33905510 PMC8262712

[ref25] Lai M. J. Lin N. T. Hu A. Soo P. C. Chen L. K. Chen L. H.. (2011). Antibacterial activity of *Acinetobacter baumannii* phage ϕAB2 endolysin (LysAB2) against both gram-positive and gram-negative bacteria. Appl. Microbiol. Biotechnol. 90, 529–539. doi: 10.1007/s00253-011-3104-y, PMID: 21264466

[ref26] Lin Z. Akin H. Rao R. Hie B. Zhu Z. Lu W.. (2023). Evolutionary-scale prediction of atomic-level protein structure with a language model. Science 379, 1123–1130. doi: 10.1126/science.ade2574 36927031

[ref27] Liu H. Kheirvari M. Tumban E. (2023). Potential applications of thermophilic bacteriophages in one health. Int. J. Mol. Sci. 24:222. doi: 10.3390/ijms24098222 PMC10179064 37175929

[ref28] Lood R. Winer B. Y. Pelzek A. J. Diez-Martinez R. Thandar M. Euler C. W.. (2015). Novel phage lysin capable of killing the multidrug-resistant gram-negative bacterium *Acinetobacter baumannii* in a mouse bacteremia model. Antimicrob. Agents Chemother. 59, 1983–1991. doi: 10.1128/AAC.04641-14, PMID: 25605353 PMC4356752

[ref29] Matsushita I. Yanase H. (2008). A novel thermophilic lysozyme from bacteriophage phiIN93. Biochem. Biophys. Res. Commun. 377, 89–92. doi: 10.1016/j.bbrc.2008.09.101, PMID: 18831965

[ref30] Mirdita M. Schütze K. Moriwaki Y. Heo L. Ovchinnikov S. Steinegger M. (2022). ColabFold: making protein folding accessible to all. Nat. Methods 19, 679–682. doi: 10.1038/s41592-022-01488-1, PMID: 35637307 PMC9184281

[ref31] Nandi A. Yadav R. Singh A. (2022). Phage derived lytic peptides, a secret weapon against *Acinetobacter baumannii*—an *in silico* approach. Front. Med. 9:1047752. doi: 10.3389/fmed.2022.1047752, PMID: 36405598 PMC9672511

[ref32] Nelson D. Loomis L. Fischetti V. A. (2001). Prevention and elimination of upper respiratory colonization of mice by group a streptococci by using a bacteriophage lytic enzyme. Proc. Natl. Acad. Sci. U. S. A. 98, 4107–4112. doi: 10.1073/pnas.061038398, PMID: 11259652 PMC31187

[ref33] Panigrahi S. K. Desiraju G. R. (2007). Strong and weak hydrogen bonds in the protein-ligand interface. Proteins 67, 128–141. doi: 10.1002/prot.21253 17206656

[ref34] Panuszko A. Bruździak P. Zielkiewicz J. Wyrzykowski D. Stangret J. (2009). Effects of urea and trimethylamine-N-oxide on the properties of water and the secondary structure of hen egg white lysozyme. J. Phys. Chem. B 113, 14797–14809. doi: 10.1021/jp904001m, PMID: 19813739

[ref35] Peng S. Y. You R. I. Lai M. J. Lin N. T. Chen L. K. Chang K. C. (2017). Highly potent antimicrobial modified peptides derived from the *Acinetobacter baumannii* phage endolysin LysAB2. Sci. Rep. 7:11477. doi: 10.1038/s41598-017-11832-7 28904355 PMC5597585

[ref36] Pettersen E. F. Goddard T. D. Huang C. C. Couch G. S. Greenblatt D. M. Meng E. C.. (2004). UCSF chimera--a visualization system for exploratory research and analysis. J. Comput. Chem. 25, 1605–1612. doi: 10.1002/jcc.20084, PMID: 15264254

[ref37] Philo J. S. (2011). Limiting the sedimentation coefficient for sedimentation velocity data analysis: partial boundary modeling and g(s) approaches revisited. Anal. Biochem. 412, 189–202. doi: 10.1016/j.ab.2011.01.035, PMID: 21284932

[ref38] Plotka M. Kaczorowska A. K. Morzywolek A. Makowska J. Kozlowski L. P. Thorisdottir A.. (2015). Biochemical characterization and validation of a catalytic site of a highly thermostable Ts2631 Endolysin from the *Thermus scotoductus* phage vB_Tsc2631. PloS One 10:e0137374. doi: 10.1371/journal.pone.0137374, PMID: 26375388 PMC4573324

[ref39] Plotka M. Kaczorowska A. K. Stefanska A. Morzywolek A. Fridjonsson O. H. Dunin-Horkawicz S.. (2014). Novel highly thermostable endolysin from *Thermus scotoductus* MAT2119 bacteriophage Ph2119 with amino acid sequence similarity to eukaryotic peptidoglycan recognition proteins. Appl. Environ. Microbiol. 80, 886–895. doi: 10.1128/AEM.03074-13, PMID: 24271162 PMC3911187

[ref40] Plotka M. Kapusta M. Dorawa S. Kaczorowska A. K. Kaczorowski T. (2019). Ts2631 Endolysin from the Extremophilic *Thermus scotoductus* bacteriophage vB_Tsc2631 as an antimicrobial agent against gram-negative multidrug-resistant Bacteria. Viruses 11:657. doi: 10.3390/v11070657, PMID: 31323845 PMC6669862

[ref41] Plotka M. Sancho-Vaello E. Dorawa S. Kaczorowska A. K. Kozlowski L. P. Kaczorowski T.. (2019). Structure and function of the Ts2631 endolysin of *Thermus scotoductus* phage vB_Tsc2631 with unique N-terminal extension used for peptidoglycan binding. Sci. Rep. 9:1261. doi: 10.1038/s41598-018-37417-6, PMID: 30718611 PMC6361986

[ref42] Plotka M. Szadkowska M. Håkansson M. Kovačič R. Al-Karadaghi S. Walse B.. (2020). Molecular characterization of a novel lytic enzyme LysC from *Clostridium intestinale* URNW and its antibacterial activity mediated by positively charged N-terminal extension. Int. J. Mol. Sci. 21:4894. doi: 10.3390/ijms21144894, PMID: 32664473 PMC7404271

[ref43] Premetis G. E. Georgakis N. D. Stathi A. Labrou N. E. (2023). Metaviromics analysis of marine biofilm reveals a glycoside hydrolase endolysin with high specificity towards *Acinetobacter baumannii*. Biochim. Biophys. Acta Proteins Proteomics 1871:140918. doi: 10.1016/j.bbapap.2023.140918 37150474

[ref44] Quintela J. C. Pittenauer E. Allmaier G. Arán V. de Pedro M. A. (1995). Structure of peptidoglycan from *Thermus thermophilus* HB8. J. Bacteriol. 177, 4947–4962. doi: 10.1128/jb.177.17.4947-4962.1995, PMID: 7665471 PMC177270

[ref45] Raz A. Serrano A. Hernandez A. Euler C. W. Fischetti V. A. (2019). Isolation of phage Lysins that effectively kill *Pseudomonas aeruginosa* in mouse models of lung and skin infection. Antimicrob. Agents Chemother. 63:19. doi: 10.1128/AAC.00024-19, PMID: 31010858 PMC6591642

[ref46] Sancho-Vaello E. Gil-Carton D. François P. Bonetti E. J. Kreir M. Pothula K. R.. (2020). The structure of the antimicrobial human cathelicidin LL-37 shows oligomerization and channel formation in the presence of membrane mimics. Sci. Rep. 10:17356. doi: 10.1038/s41598-020-74401-5, PMID: 33060695 PMC7562864

[ref47] Sarkar T. Chetia M. Chatterjee S. (2021). Antimicrobial peptides and proteins: from Nature’s reservoir to the laboratory and beyond. Front. Chem. 9:691532. doi: 10.3389/fchem.2021.691532, PMID: 34222199 PMC8249576

[ref48] Schleifer K. H. Kandler O. (1972). Peptidoglycan types of bacterial cell walls and their taxonomic implications. Bacteriol. Rev. 36, 407–477. doi: 10.1128/br.36.4.407-477.1972, PMID: 4568761 PMC408328

[ref49] Schmelcher M. Donovan D. M. Loessner M. J. (2012). Bacteriophage endolysins as novel antimicrobials. Future Microbiol. 7, 1147–1171. doi: 10.2217/fmb.12.97, PMID: 23030422 PMC3563964

[ref50] Schuch R. M. L. Hoffenberg S. Wittekind M. (2023). LYSIN polypeptides active against GRAM-negative BACTERIA. United States patent no. 20230050560.

[ref51] Schuck P. (2000). Size-distribution analysis of macromolecules by sedimentation velocity ultracentrifugation and lamm equation modeling. Biophys. J. 78, 1606–1619. doi: 10.1016/S0006-3495(00)76713-0, PMID: 10692345 PMC1300758

[ref52] Sievers F. Wilm A. Dineen D. Gibson T. J. Karplus K. Li W.. (2011). Fast, scalable generation of high-quality protein multiple sequence alignments using Clustal omega. Mol. Syst. Biol. 7:539. doi: 10.1038/msb.2011.75, PMID: 21988835 PMC3261699

[ref53] Son S. M. Kim J. Ryu S. (2023). Development of sensitizer peptide-fused endolysin Lys1S-L9P acting against multidrug-resistant gram-negative bacteria. Front. Microbiol. 14:6796. doi: 10.3389/fmicb.2023.1296796, PMID: 38075915 PMC10701683

[ref54] Son B. Yun J. Lim J. A. Shin H. Heu S. Ryu S. (2012). Characterization of LysB4, an endolysin from the *Bacillus cereus*-infecting bacteriophage B4. BMC Microbiol. 12:33. doi: 10.1186/1471-2180-12-33 22416675 PMC3315420

[ref55] Szadkowska M. Olewniczak M. Kloska A. Jankowska E. Kapusta M. Rybak B.. (2022). A novel cryptic Clostridial peptide that kills Bacteria by a cell membrane Permeabilization mechanism. Microbiol Spectr 10:e0165722. doi: 10.1128/spectrum.01657-22 36094301 PMC9602519

[ref56] Tacconelli E. Carrara E. Savoldi A. Harbarth S. Mendelson M. Monnet D. L.. (2018). Discovery, research, and development of new antibiotics: the WHO priority list of antibiotic-resistant bacteria and tuberculosis. Lancet Infect. Dis. 18, 318–327. doi: 10.1016/S1473-3099(17)30753-3 29276051

[ref57] Thomas S. Karnik S. Barai R. S. Jayaraman V. K. Idicula-Thomas S. (2010). CAMP: a useful resource for research on antimicrobial peptides. Nucleic Acids Res. 38, D774–D780. doi: 10.1093/nar/gkp1021, PMID: 19923233 PMC2808926

[ref58] Vermassen A. Leroy S. Talon R. Provot C. Popowska M. Desvaux M. (2019). Cell Wall hydrolases in Bacteria: insight on the diversity of Cell Wall amidases, Glycosidases and peptidases toward peptidoglycan. Front. Microbiol. 10:331. doi: 10.3389/fmicb.2019.00331, PMID: 30873139 PMC6403190

[ref59] Vollmer W. Blanot D. de Pedro M. A. (2008). Peptidoglycan structure and architecture. FEMS Microbiol. Rev. 32, 149–167. doi: 10.1111/j.1574-6976.2007.00094.x 18194336

[ref60] Vorland L. H. Ulvatne H. Rekdal O. Svendsen J. S. (1999). Initial binding sites of antimicrobial peptides in *Staphylococcus aureus* and *Escherichia coli*. Scand. J. Infect. Dis. 31, 467–473. doi: 10.1080/00365549950163987, PMID: 10576125

[ref61] Wang F. Ji X. Li Q. Zhang G. Peng J. Hai J.. (2020). TSPphg Lysin from the Extremophilic *Thermus* bacteriophage TSP4 as a potential antimicrobial agent against both gram-negative and gram-positive pathogenic Bacteria. Viruses 12:192. doi: 10.3390/v12020192 32050494 PMC7077265

[ref62] Wang G. Li X. Wang Z. (2016). APD3: the antimicrobial peptide database as a tool for research and education. Nucleic Acids Res. 44, D1087–D1093. doi: 10.1093/nar/gkv1278, PMID: 26602694 PMC4702905

[ref63] Wang F. Xiong Y. Xiao Y. Han J. Deng X. Lin L. (2020). MMPphg from the thermophilic Meiothermus bacteriophage MMP17 as a potential antimicrobial agent against both gram-negative and gram-positive bacteria. Virol. J. 17:130. doi: 10.1186/s12985-020-01403-0, PMID: 32843096 PMC7448439

[ref64] Ye T. Zhang X. (2008). Characterization of a lysin from deep-sea thermophilic bacteriophage GVE2. Appl. Microbiol. Biotechnol. 78, 635–641. doi: 10.1007/s00253-008-1353-1, PMID: 18224315

[ref65] Zampara A. Sørensen M. C. H. Grimon D. Antenucci F. Vitt A. R. Bortolaia V.. (2020). Exploiting phage receptor binding proteins to enable endolysins to kill gram-negative bacteria. Sci. Rep. 10:12087. doi: 10.1038/s41598-020-68983-3, PMID: 32694655 PMC7374709

[ref66] Żebrowska J. Żołnierkiewicz O. Ponikowska M. Puchalski M. Krawczun N. Makowska J.. (2022). Cloning and characterization of a thermostable Endolysin of bacteriophage TP-84 as a potential disinfectant and biofilm-removing biological agent. Int. J. Mol. Sci. 23:612. doi: 10.3390/ijms23147612, PMID: 35886960 PMC9325043

[ref67] Zeth K. Sancho-Vaello E. (2021). Structural plasticity of LL-37 indicates elaborate functional adaptation mechanisms to bacterial target structures. Int. J. Mol. Sci. 22:5200. doi: 10.3390/ijms22105200, PMID: 34068993 PMC8156758

