# Supplementary material for: Molecular characterization of the PhiKo endolysin from Thermus thermophilus HB27 bacteriophage phiKo and its cryptic lytic peptide RAP-29
Source: Front Microbiol. 2024 Jan 19;14:1303794. doi: 10.3389/fmicb.2023.1303794 (PMC10836841; doi:10.3389/fmicb.2023.1303794)
Supplement: Supplementary file 1 [file Data_Sheet_1.docx]

Supplementary Material

# Supplementary Data

# Supplementary Figures and Tables

## Supplementary Tables

**Supplementary Table 1.** Bactericidal activity of PhiKo protein against Gram-positive and Gram-negative bacteria.

| **No.** | **Bacterial Species** | **Bactericidal activity**  **[log reduction]** |
| --- | --- | --- |
| Gram-positive bacteria | | |
| 1. | Staphylococcus aureus ATCC 25923 | 0.82 ± 0.05 |
| 2. | *Staphylococcus aureus* KPD 425 | 0.75 ± 0.05 |
| 3. | *Staphylococcus aureus* KPD 740 | 0.0 |
| 4. | *Staphylococcus epidermidis* KPD 440 | 0.0 |
| 5. | *Staphylococcus hominis* KPD 910 | 0.0 |
| 6. | *Staphylococcus pettenkoferi* KPD 741 | 4.40 ± 0.09 |
| Gram-negative bacteria | | |
| 7. | *Acinetobacter baumannii* CRAB KPD 205 | 2.57 ± 0.07 |
| 8. | *Acinetobacter baumannii* MDR KPD 581 | 0.44 ± 0.10 |
| 9. | *Acinetobacter baumannii* KPD 735 BA | 0.0 |
| 10. | *Enterobacter cloaceae*  KPD 297 | 0.30 ± 0.02 |
| 11. | *Escherichia coli* KPD 217 | 0.0 |
| 12. | *Klebsiella pneumoniae* KPD 298 | 0.0 |
| 13. | *Pseudomonas aeruginosa* KPD 430 | 0.58 ± 0.10 |
| 14. | *Pseudomonas aeruginosa* CRPA KPD 431 | 0.62 ± 0.08 |
| 15. | *Pseudomonas aeruginosa* KPD PAO 1 | 0.61 ± 0.05 |

**Supplementary Table** **2.** Prediction of antimicrobial regions within PhiKo endolysin sequence.

| **Classifier:** | | | | | | **SVM** | |  | **RF** | | | | **ANN** | **DA** | |
| --- | --- | --- | --- | --- | --- | --- | --- | --- | --- | --- | --- | --- | --- | --- | --- |
| **Seq. ID.** | **Position** | | **Sequence** | | **Class** | | **AMP**  **probability** | | **Class** | | **AMP**  **probability** | | **Class** | **Class** | **AMP**  **probability** |
|  | 1-29 | MNWIEFWRSKKPTWRHRPVDPAYIVLHHT | | NAMP | | | 0.010 | |  | NAMP | | 0.029 | NAMP | NAMP | 0.001 |
|  | 2-30 | NWIEFWRSKKPTWRHRPVDPAYIVLHHTA | | NAMP | | | 0.080 | |  | NAMP | | 0.094 | NAMP | NAMP | 0.067 |
|  | 3-31 | WIEFWRSKKPTWRHRPVDPAYIVLHHTAG | | NAMP | | | 0.087 | |  | NAMP | | 0.153 | NAMP | NAMP | 0.076 |
|  | 4-32 | IEFWRSKKPTWRHRPVDPAYIVLHHTAGP | | NAMP | | | 0.096 | |  | NAMP | | 0.124 | NAMP | NAMP | 0.047 |
|  | 5-33 | EFWRSKKPTWRHRPVDPAYIVLHHTAGPV | | NAMP | | | 0.102 | |  | NAMP | | 0.149 | NAMP | NAMP | 0.044 |
|  | 6-34 | FWRSKKPTWRHRPVDPAYIVLHHTAGPVD | | NAMP | | | 0.086 | |  | NAMP | | 0.160 | NAMP | NAMP | 0.037 |
|  | 7-35 | WRSKKPTWRHRPVDPAYIVLHHTAGPVDQ | | NAMP | | | 0.046 | |  | NAMP | | 0.075 | NAMP | NAMP | 0.024 |
|  | 8-36 | RSKKPTWRHRPVDPAYIVLHHTAGPVDQA | | NAMP | | | 0.047 | |  | NAMP | | 0.069 | NAMP | NAMP | 0.015 |
|  | 9-37 | SKKPTWRHRPVDPAYIVLHHTAGPVDQAP | | NAMP | | | 0.036 | |  | NAMP | | 0.032 | NAMP | NAMP | 0.006 |
|  | 10-38 | KKPTWRHRPVDPAYIVLHHTAGPVDQAPQ | | NAMP | | | 0.049 | |  | NAMP | | 0.028 | NAMP | NAMP | 0.016 |
|  | 11-39 | KPTWRHRPVDPAYIVLHHTAGPVDQAPQA | | NAMP | | | 0.044 | |  | NAMP | | 0.001 | NAMP | NAMP | 0.007 |
|  | 12-40 | PTWRHRPVDPAYIVLHHTAGPVDQAPQAI | | NAMP | | | 0.041 | |  | NAMP | | 0.005 | NAMP | NAMP | 0.019 |
|  | 13-41 | TWRHRPVDPAYIVLHHTAGPVDQAPQAIW | | NAMP | | | 0.031 | |  | NAMP | | 0.002 | NAMP | NAMP | 0.028 |
|  | 14-42 | WRHRPVDPAYIVLHHTAGPVDQAPQAIWD | | NAMP | | | 0.021 | |  | NAMP | | 0.007 | NAMP | NAMP | 0.011 |
|  | 15-43 | RHRPVDPAYIVLHHTAGPVDQAPQAIWDY | | NAMP | | | 0.032 | |  | NAMP | | 0.002 | NAMP | NAMP | 0.012 |
|  | 16-44 | HRPVDPAYIVLHHTAGPVDQAPQAIWDYH | | NAMP | | | 0.019 | |  | NAMP | | 0.004 | NAMP | NAMP | 0.007 |
|  | 17-45 | RPVDPAYIVLHHTAGPVDQAPQAIWDYHV | | NAMP | | | 0.033 | |  | NAMP | | 0.016 | NAMP | NAMP | 0.025 |
|  | 18-46 | PVDPAYIVLHHTAGPVDQAPQAIWDYHVK | | NAMP | | | 0.078 | |  | NAMP | | 0.039 | NAMP | NAMP | 0.114 |
|  | 19-47 | VDPAYIVLHHTAGPVDQAPQAIWDYHVKV | | NAMP | | | 0.098 | |  | NAMP | | 0.091 | NAMP | NAMP | 0.165 |
|  | 20-48 | DPAYIVLHHTAGPVDQAPQAIWDYHVKVR | | NAMP | | | 0.058 | |  | NAMP | | 0.007 | NAMP | NAMP | 0.052 |
|  | 21-49 | PAYIVLHHTAGPVDQAPQAIWDYHVKVRG | | NAMP | | | 0.091 | |  | NAMP | | 0.021 | NAMP | NAMP | 0.081 |
|  | 22-50 | AYIVLHHTAGPVDQAPQAIWDYHVKVRGW | | NAMP | | | 0.082 | |  | NAMP | | 0.072 | NAMP | NAMP | 0.037 |
|  | 23-51 | YIVLHHTAGPVDQAPQAIWDYHVKVRGWP | | NAMP | | | 0.048 | |  | NAMP | | 0.021 | NAMP | NAMP | 0.031 |
|  | 24-52 | IVLHHTAGPVDQAPQAIWDYHVKVRGWPH | | NAMP | | | 0.079 | |  | NAMP | | 0.034 | NAMP | NAMP | 0.091 |
|  | 25-53 | VLHHTAGPVDQAPQAIWDYHVKVRGWPHG | | NAMP | | | 0.072 | |  | NAMP | | 0.030 | NAMP | NAMP | 0.045 |
|  | 26-54 | LHHTAGPVDQAPQAIWDYHVKVRGWPHGG | | NAMP | | | 0.076 | |  | NAMP | | 0.029 | NAMP | NAMP | 0.033 |
|  | 27-55 | HHTAGPVDQAPQAIWDYHVKVRGWPHGGY | | NAMP | | | 0.050 | |  | NAMP | | 0.009 | NAMP | NAMP | 0.030 |
|  | 28-56 | HTAGPVDQAPQAIWDYHVKVRGWPHGGYH | | NAMP | | | 0.058 | |  | NAMP | | 0.020 | NAMP | NAMP | 0.038 |
|  | 29-57 | TAGPVDQAPQAIWDYHVKVRGWPHGGYHF | | NAMP | | | 0.211 | |  | NAMP | | 0.042 | NAMP | NAMP | 0.053 |
|  | 30-58 | AGPVDQAPQAIWDYHVKVRGWPHGGYHFL | | NAMP | | | 0.247 | |  | NAMP | | 0.136 | NAMP | NAMP | 0.062 |
|  | 31-59 | GPVDQAPQAIWDYHVKVRGWPHGGYHFLV | | NAMP | | | 0.221 | |  | NAMP | | 0.109 | NAMP | NAMP | 0.075 |
|  | 32-60 | PVDQAPQAIWDYHVKVRGWPHGGYHFLVY | | NAMP | | | 0.086 | |  | NAMP | | 0.062 | NAMP | NAMP | 0.036 |
|  | 33-61 | VDQAPQAIWDYHVKVRGWPHGGYHFLVYH | | NAMP | | | 0.085 | |  | NAMP | | 0.084 | NAMP | NAMP | 0.075 |
|  | 34-62 | DQAPQAIWDYHVKVRGWPHGGYHFLVYHD | | NAMP | | | 0.032 | |  | NAMP | | 0.035 | NAMP | NAMP | 0.027 |
|  | 35-63 | QAPQAIWDYHVKVRGWPHGGYHFLVYHDG | | NAMP | | | 0.052 | |  | NAMP | | 0.034 | NAMP | NAMP | 0.024 |
|  | 36-64 | APQAIWDYHVKVRGWPHGGYHFLVYHDGT | | NAMP | | | 0.049 | |  | NAMP | | 0.069 | NAMP | NAMP | 0.030 |
|  | 37-65 | PQAIWDYHVKVRGWPHGGYHFLVYHDGTV | | NAMP | | | 0.027 | |  | NAMP | | 0.044 | NAMP | NAMP | 0.012 |
|  | 38-66 | QAIWDYHVKVRGWPHGGYHFLVYHDGTVV | | NAMP | | | 0.039 | |  | NAMP | | 0.100 | NAMP | NAMP | 0.029 |
|  | 39-67 | AIWDYHVKVRGWPHGGYHFLVYHDGTVVK | | NAMP | | | 0.053 | |  | NAMP | | 0.134 | NAMP | NAMP | 0.066 |
|  | 40-68 | IWDYHVKVRGWPHGGYHFLVYHDGTVVKM | | NAMP | | | 0.027 | |  | NAMP | | 0.034 | NAMP | NAMP | 0.020 |
|  | 41-69 | WDYHVKVRGWPHGGYHFLVYHDGTVVKML | | NAMP | | | 0.016 | |  | NAMP | | 0.021 | NAMP | NAMP | 0.012 |
|  | 42-70 | DYHVKVRGWPHGGYHFLVYHDGTVVKMLP | | NAMP | | | 0.030 | |  | NAMP | | 0.017 | NAMP | NAMP | 0.011 |
|  | 43-71 | YHVKVRGWPHGGYHFLVYHDGTVVKMLPL | | NAMP | | | 0.027 | |  | NAMP | | 0.136 | NAMP | NAMP | 0.013 |
|  | 44-72 | HVKVRGWPHGGYHFLVYHDGTVVKMLPLS | | NAMP | | | 0.041 | |  | NAMP | | 0.125 | NAMP | NAMP | 0.034 |
|  | 45-73 | VKVRGWPHGGYHFLVYHDGTVVKMLPLSA | | NAMP | | | 0.146 | |  | NAMP | | 0.269 | NAMP | NAMP | 0.070 |
|  | 46-74 | KVRGWPHGGYHFLVYHDGTVVKMLPLSAQ | | NAMP | | | 0.126 | |  | NAMP | | 0.080 | NAMP | NAMP | 0.051 |
|  | 47-75 | VRGWPHGGYHFLVYHDGTVVKMLPLSAQP | | NAMP | | | 0.078 | |  | NAMP | | 0.029 | NAMP | NAMP | 0.044 |
|  | 48-76 | RGWPHGGYHFLVYHDGTVVKMLPLSAQPI | | NAMP | | | 0.111 | |  | NAMP | | 0.043 | NAMP | NAMP | 0.064 |
|  | 49-77 | GWPHGGYHFLVYHDGTVVKMLPLSAQPIC | | NAMP | | | 0.177 | |  | NAMP | | 0.030 | NAMP | NAMP | 0.042 |
|  | 50-78 | WPHGGYHFLVYHDGTVVKMLPLSAQPICV | | NAMP | | | 0.087 | |  | NAMP | | 0.013 | NAMP | NAMP | 0.014 |
|  | 51-79 | PHGGYHFLVYHDGTVVKMLPLSAQPICVG | | NAMP | | | 0.198 | |  | NAMP | | 0.051 | NAMP | NAMP | 0.030 |
|  | 52-80 | HGGYHFLVYHDGTVVKMLPLSAQPICVGE | | NAMP | | | 0.087 | |  | NAMP | | 0.036 | NAMP | NAMP | 0.016 |
|  | 53-81 | GGYHFLVYHDGTVVKMLPLSAQPICVGEY | | NAMP | | | 0.171 | |  | NAMP | | 0.048 | NAMP | NAMP | 0.018 |
|  | 54-82 | GYHFLVYHDGTVVKMLPLSAQPICVGEYN | | NAMP | | | 0.080 | |  | NAMP | | 0.018 | NAMP | NAMP | 0.014 |
|  | 55-83 | YHFLVYHDGTVVKMLPLSAQPICVGEYNH | | NAMP | | | 0.023 | |  | NAMP | | 0.005 | NAMP | NAMP | 0.004 |
|  | 56-84 | HFLVYHDGTVVKMLPLSAQPICVGEYNHL | | NAMP | | | 0.029 | |  | NAMP | | 0.000 | NAMP | NAMP | 0.012 |
|  | 57-85 | FLVYHDGTVVKMLPLSAQPICVGEYNHLA | | NAMP | | | 0.081 | |  | NAMP | | 0.013 | NAMP | NAMP | 0.027 |
|  | 58-86 | LVYHDGTVVKMLPLSAQPICVGEYNHLAI | | NAMP | | | 0.109 | |  | NAMP | | 0.033 | NAMP | NAMP | 0.010 |
|  | 59-87 | VYHDGTVVKMLPLSAQPICVGEYNHLAIC | | NAMP | | | 0.168 | |  | NAMP | | 0.016 | NAMP | NAMP | 0.038 |
|  | 60-88 | YHDGTVVKMLPLSAQPICVGEYNHLAICI | | NAMP | | | 0.124 | |  | NAMP | | 0.029 | NAMP | NAMP | 0.018 |
|  | 61-89 | HDGTVVKMLPLSAQPICVGEYNHLAICIA | | NAMP | | | 0.211 | |  | NAMP | | 0.076 | NAMP | NAMP | 0.022 |
|  | 62-90 | DGTVVKMLPLSAQPICVGEYNHLAICIAL | | AMP | | | 0.605 | |  | NAMP | | 0.250 | NAMP | NAMP | 0.211 |
|  | 63-91 | GTVVKMLPLSAQPICVGEYNHLAICIALV | | AMP | | | 0.730 | |  | AMP | | 0.535 | AMP | NAMP | 0.315 |
|  | 64-92 | TVVKMLPLSAQPICVGEYNHLAICIALVG | | AMP | | | 0.539 | |  | NAMP | | 0.349 | NAMP | NAMP | 0.220 |
|  | 65-93 | VVKMLPLSAQPICVGEYNHLAICIALVGN | | AMP | | | 0.665 | |  | AMP | | 0.548 | AMP | NAMP | 0.480 |
|  | 66-94 | VKMLPLSAQPICVGEYNHLAICIALVGNF | | AMP | | | 0.553 | |  | NAMP | | 0.477 | NAMP | NAMP | 0.253 |
|  | 67-95 | KMLPLSAQPICVGEYNHLAICIALVGNFV | | NAMP | | | 0.409 | |  | NAMP | | 0.205 | NAMP | NAMP | 0.236 |
|  | 68-96 | MLPLSAQPICVGEYNHLAICIALVGNFVG | | NAMP | | | 0.352 | |  | NAMP | | 0.140 | NAMP | NAMP | 0.135 |
|  | 69-97 | LPLSAQPICVGEYNHLAICIALVGNFVGG | | AMP | | | 0.884 | |  | AMP | | 0.680 | AMP | AMP | 0.694 |
|  | 70-98 | PLSAQPICVGEYNHLAICIALVGNFVGGY | | AMP | | | 0.816 | |  | NAMP | | 0.454 | AMP | AMP | 0.709 |
|  | **71-99** | **LSAQPICVGEYNHLAICIALVGNFVGGYP** | | **AMP** | | | **0.840** | |  | **AMP** | | **0.624** | **AMP** | **AMP** | **0.615** |
|  | 72-100 | SAQPICVGEYNHLAICIALVGNFVGGYPP | | AMP | | | 0.784 | |  | NAMP | | 0.476 | AMP | AMP | 0.560 |
|  | 73-101 | AQPICVGEYNHLAICIALVGNFVGGYPPE | | AMP | | | 0.608 | |  | NAMP | | 0.358 | NAMP | NAMP | 0.476 |
|  | 74-102 | QPICVGEYNHLAICIALVGNFVGGYPPEW | | NAMP | | | 0.311 | |  | NAMP | | 0.239 | NAMP | NAMP | 0.124 |
|  | 75-103 | PICVGEYNHLAICIALVGNFVGGYPPEWN | | AMP | | | 0.511 | |  | NAMP | | 0.255 | NAMP | NAMP | 0.232 |
|  | 76-104 | ICVGEYNHLAICIALVGNFVGGYPPEWNE | | NAMP | | | 0.431 | |  | NAMP | | 0.338 | NAMP | NAMP | 0.499 |
|  | 77-105 | CVGEYNHLAICIALVGNFVGGYPPEWNER | | NAMP | | | 0.258 | |  | NAMP | | 0.242 | NAMP | NAMP | 0.325 |
|  | 78-106 | VGEYNHLAICIALVGNFVGGYPPEWNERA | | NAMP | | | 0.233 | |  | NAMP | | 0.209 | NAMP | NAMP | 0.196 |
|  | 79-107 | GEYNHLAICIALVGNFVGGYPPEWNERAP | | NAMP | | | 0.224 | |  | NAMP | | 0.116 | NAMP | NAMP | 0.084 |
|  | 80-108 | EYNHLAICIALVGNFVGGYPPEWNERAPG | | NAMP | | | 0.124 | |  | NAMP | | 0.069 | NAMP | NAMP | 0.020 |
|  | 81-109 | YNHLAICIALVGNFVGGYPPEWNERAPGW | | NAMP | | | 0.114 | |  | NAMP | | 0.124 | NAMP | NAMP | 0.022 |
|  | 82-110 | NHLAICIALVGNFVGGYPPEWNERAPGWK | | NAMP | | | 0.155 | |  | NAMP | | 0.157 | NAMP | NAMP | 0.111 |
|  | 83-111 | HLAICIALVGNFVGGYPPEWNERAPGWKS | | NAMP | | | 0.161 | |  | NAMP | | 0.133 | NAMP | NAMP | 0.076 |
|  | 84-112 | LAICIALVGNFVGGYPPEWNERAPGWKSL | | AMP | | | 0.516 | |  | AMP | | 0.523 | AMP | NAMP | 0.252 |
|  | 85-113 | AICIALVGNFVGGYPPEWNERAPGWKSLA | | AMP | | | 0.534 | |  | AMP | | 0.534 | AMP | NAMP | 0.458 |
|  | 86-114 | ICIALVGNFVGGYPPEWNERAPGWKSLAW | | AMP | | | 0.549 | |  | AMP | | 0.503 | AMP | NAMP | 0.299 |
|  | 87-115 | CIALVGNFVGGYPPEWNERAPGWKSLAWL | | NAMP | | | 0.398 | |  | NAMP | | 0.469 | AMP | NAMP | 0.227 |
|  | 88-116 | IALVGNFVGGYPPEWNERAPGWKSLAWLV | | NAMP | | | 0.359 | |  | NAMP | | 0.432 | NAMP | NAMP | 0.378 |
|  | 89-117 | ALVGNFVGGYPPEWNERAPGWKSLAWLVR | | NAMP | | | 0.260 | |  | NAMP | | 0.299 | NAMP | NAMP | 0.359 |
|  | 90-118 | LVGNFVGGYPPEWNERAPGWKSLAWLVRE | | NAMP | | | 0.128 | |  | NAMP | | 0.203 | NAMP | NAMP | 0.069 |
|  | 91-119 | VGNFVGGYPPEWNERAPGWKSLAWLVREL | | NAMP | | | 0.241 | |  | NAMP | | 0.205 | NAMP | NAMP | 0.157 |
|  | 92-120 | GNFVGGYPPEWNERAPGWKSLAWLVRELR | | NAMP | | | 0.263 | |  | NAMP | | 0.162 | NAMP | NAMP | 0.287 |
|  | 93-121 | NFVGGYPPEWNERAPGWKSLAWLVRELRK | | NAMP | | | 0.300 | |  | NAMP | | 0.176 | NAMP | NAMP | 0.209 |
|  | 94-122 | FVGGYPPEWNERAPGWKSLAWLVRELRKH | | NAMP | | | 0.262 | |  | NAMP | | 0.109 | NAMP | NAMP | 0.085 |
|  | 95-123 | VGGYPPEWNERAPGWKSLAWLVRELRKHD | | NAMP | | | 0.186 | |  | NAMP | | 0.066 | NAMP | NAMP | 0.049 |
|  | 96-124 | GGYPPEWNERAPGWKSLAWLVRELRKHDS | | NAMP | | | 0.238 | |  | NAMP | | 0.060 | NAMP | NAMP | 0.049 |
|  | 97-125 | GYPPEWNERAPGWKSLAWLVRELRKHDSG | | NAMP | | | 0.209 | |  | NAMP | | 0.052 | NAMP | NAMP | 0.035 |
|  | 98-126 | YPPEWNERAPGWKSLAWLVRELRKHDSGL | | NAMP | | | 0.123 | |  | NAMP | | 0.040 | NAMP | NAMP | 0.004 |
|  | 99-127 | PPEWNERAPGWKSLAWLVRELRKHDSGLR | | NAMP | | | 0.185 | |  | NAMP | | 0.092 | NAMP | NAMP | 0.066 |
|  | 100-128 | PEWNERAPGWKSLAWLVRELRKHDSGLRL | | NAMP | | | 0.323 | |  | NAMP | | 0.190 | NAMP | NAMP | 0.086 |
|  | 101-129 | EWNERAPGWKSLAWLVRELRKHDSGLRLR | | AMP | | | 0.634 | |  | NAMP | | 0.468 | NAMP | NAMP | 0.352 |
|  | **102-130** | **WNERAPGWKSLAWLVRELRKHDSGLRLRL** | | **AMP** | | | **0.830** | |  | **AMP** | | **0.654** | **AMP** | **AMP** | **0.712** |
|  | **103-131** | **NERAPGWKSLAWLVRELRKHDSGLRLRLV** | | **AMP** | | | **0.799** | |  | **AMP** | | **0.635** | **AMP** | **AMP** | **0.756** |
|  | **104-132** | **ERAPGWKSLAWLVRELRKHDSGLRLRLVR** | | **AMP** | | | **0.906** | |  | **AMP** | | **0.700** | **AMP** | **AMP** | **0.797** |
|  | **105-133** | **RAPGWKSLAWLVRELRKHDSGLRLRLVRH** | | **AMP** | | | **0.933** | |  | **AMP** | | **0.714** | **AMP** | **AMP** | **0.817** |
|  | **106-134** | **APGWKSLAWLVRELRKHDSGLRLRLVRHK** | | **AMP** | | | **0.867** | |  | **AMP** | | **0.654** | **AMP** | **AMP** | **0.779** |
|  | 107-135 | PGWKSLAWLVRELRKHDSGLRLRLVRHKD | | AMP | | | 0.770 | |  | AMP | | 0.622 | NAMP | AMP | 0.601 |
|  | **108-136** | **GWKSLAWLVRELRKHDSGLRLRLVRHKDL** | | **AMP** | | | **0.925** | |  | **AMP** | | **0.700** | **AMP** | **AMP** | **0.948** |
|  | 109-137 | WKSLAWLVRELRKHDSGLRLRLVRHKDLR | | AMP | | | 0.907 | |  | AMP | | 0.689 | NAMP | AMP | 0.799 |
|  | **110-138** | **KSLAWLVRELRKHDSGLRLRLVRHKDLRP** | | **AMP** | | | **0.840** | |  | **AMP** | | **0.671** | **AMP** | **AMP** | **0.583** |
|  | 111-139 | SLAWLVRELRKHDSGLRLRLVRHKDLRPT | | AMP | | | 0.568 | |  | AMP | | 0.552 | NAMP | NAMP | 0.371 |
|  | **112-140** | **LAWLVRELRKHDSGLRLRLVRHKDLRPTK** | | **AMP** | | | **0.892** | |  | **AMP** | | **0.698** | **AMP** | **AMP** | **0.794** |
|  | **113-141** | **AWLVRELRKHDSGLRLRLVRHKDLRPTKC** | | **AMP** | | | **0.899** | |  | **AMP** | | **0.704** | **AMP** | **AMP** | **0.550** |
|  | 114-142 | WLVRELRKHDSGLRLRLVRHKDLRPTKCP | | AMP | | | 0.826 | |  | AMP | | 0.605 | AMP | NAMP | 0.415 |
|  | 115-143 | LVRELRKHDSGLRLRLVRHKDLRPTKCPG | | AMP | | | 0.673 | |  | AMP | | 0.579 | AMP | NAMP | 0.389 |
|  | 116-144 | VRELRKHDSGLRLRLVRHKDLRPTKCPGT | | NAMP | | | 0.395 | |  | AMP | | 0.520 | AMP | NAMP | 0.288 |
|  | 117-145 | RELRKHDSGLRLRLVRHKDLRPTKCPGTV | | NAMP | | | 0.459 | |  | AMP | | 0.535 | AMP | NAMP | 0.273 |
|  | 118-146 | ELRKHDSGLRLRLVRHKDLRPTKCPGTVT | | NAMP | | | 0.242 | |  | NAMP | | 0.453 | AMP | NAMP | 0.123 |
|  | 119-147 | LRKHDSGLRLRLVRHKDLRPTKCPGTVTW | | NAMP | | | 0.390 | |  | AMP | | 0.505 | AMP | NAMP | 0.087 |
|  | 120-148 | RKHDSGLRLRLVRHKDLRPTKCPGTVTWE | | NAMP | | | 0.222 | |  | NAMP | | 0.403 | NAMP | NAMP | 0.035 |
|  | 121-149 | KHDSGLRLRLVRHKDLRPTKCPGTVTWEE | | NAMP | | | 0.136 | |  | NAMP | | 0.227 | NAMP | NAMP | 0.007 |
|  | 122-150 | HDSGLRLRLVRHKDLRPTKCPGTVTWEEA | | NAMP | | | 0.088 | |  | NAMP | | 0.110 | NAMP | NAMP | 0.003 |
|  | 123-151 | DSGLRLRLVRHKDLRPTKCPGTVTWEEAL | | NAMP | | | 0.138 | |  | NAMP | | 0.137 | NAMP | NAMP | 0.031 |
|  | 124-152 | SGLRLRLVRHKDLRPTKCPGTVTWEEALV | | NAMP | | | 0.245 | |  | NAMP | | 0.210 | NAMP | NAMP | 0.040 |
|  | 125-153 | GLRLRLVRHKDLRPTKCPGTVTWEEALVR | | AMP | | | 0.552 | |  | NAMP | | 0.473 | AMP | NAMP | 0.236 |
|  | 126-154 | LRLRLVRHKDLRPTKCPGTVTWEEALVRG | | NAMP | | | 0.486 | |  | NAMP | | 0.452 | NAMP | NAMP | 0.072 |
|  | 127-155 | RLRLVRHKDLRPTKCPGTVTWEEALVRGG | | NAMP | | | 0.406 | |  | NAMP | | 0.428 | AMP | NAMP | 0.111 |
|  | 128-156 | LRLVRHKDLRPTKCPGTVTWEEALVRGGV | | NAMP | | | 0.341 | |  | NAMP | | 0.267 | NAMP | NAMP | 0.066 |
|  | 129-157 | RLVRHKDLRPTKCPGTVTWEEALVRGGVP | | NAMP | | | 0.235 | |  | NAMP | | 0.181 | NAMP | NAMP | 0.079 |
|  | 130-158 | LVRHKDLRPTKCPGTVTWEEALVRGGVPQ | | NAMP | | | 0.184 | |  | NAMP | | 0.141 | NAMP | NAMP | 0.022 |
|  | 131-159 | VRHKDLRPTKCPGTVTWEEALVRGGVPQE | | NAMP | | | 0.060 | |  | NAMP | | 0.063 | NAMP | NAMP | 0.005 |
|  | 132-160 | RHKDLRPTKCPGTVTWEEALVRGGVPQEQ | | NAMP | | | 0.043 | |  | NAMP | | 0.031 | NAMP | NAMP | 0.001 |
|  | 133-161 | HKDLRPTKCPGTVTWEEALVRGGVPQEQV | | NAMP | | | 0.032 | |  | NAMP | | 0.022 | NAMP | NAMP | 0.001 |
|  | 134-162 | KDLRPTKCPGTVTWEEALVRGGVPQEQVE | | NAMP | | | 0.027 | |  | NAMP | | 0.018 | NAMP | NAMP | 0.004 |
|  | 135-163 | DLRPTKCPGTVTWEEALVRGGVPQEQVET | | NAMP | | | 0.021 | |  | NAMP | | 0.018 | NAMP | NAMP | 0.002 |
|  | 136-164 | LRPTKCPGTVTWEEALVRGGVPQEQVETL | | NAMP | | | 0.032 | |  | NAMP | | 0.021 | NAMP | NAMP | 0.003 |
|  | 137-165 | RPTKCPGTVTWEEALVRGGVPQEQVETLK | | NAMP | | | 0.037 | |  | NAMP | | 0.030 | NAMP | NAMP | 0.001 |
|  | 138-166 | PTKCPGTVTWEEALVRGGVPQEQVETLKV | | NAMP | | | 0.048 | |  | NAMP | | 0.029 | NAMP | NAMP | 0.008 |
|  | 139-167 | TKCPGTVTWEEALVRGGVPQEQVETLKVA | | NAMP | | | 0.057 | |  | NAMP | | 0.028 | NAMP | NAMP | 0.005 |
|  | 140-168 | KCPGTVTWEEALVRGGVPQEQVETLKVAG | | NAMP | | | 0.105 | |  | NAMP | | 0.096 | NAMP | NAMP | 0.017 |
|  | 141-169 | CPGTVTWEEALVRGGVPQEQVETLKVAGV | | NAMP | | | 0.134 | |  | NAMP | | 0.270 | NAMP | NAMP | 0.031 |
|  | 142-170 | PGTVTWEEALVRGGVPQEQVETLKVAGVI | | NAMP | | | 0.186 | |  | NAMP | | 0.221 | NAMP | NAMP | 0.126 |
|  | 143-171 | GTVTWEEALVRGGVPQEQVETLKVAGVIA | | NAMP | | | 0.323 | |  | NAMP | | 0.308 | NAMP | NAMP | 0.457 |

Abbreviations: SVM Support Vector Machine; RF Random Forest, ANN Artificial Neural Network, and DA Discriminant Analysis; AMP antimicrobial peptide; NAMP not antimicrobial peptide.

**Supplementary Table 3.** Bacterial strains used in the study and their origin/characteristics.

| **No.** | **Bacterial Species** | **Origin/Characteristics^#^** |
| --- | --- | --- |
| Gram-positive bacteria | | |
| 1. | Staphylococcus aureus ATCC 25923 | Clinical strain (intermediate resistance to CAZ and AMO) |
| 2. | *Staphylococcus aureus* KPD 425 | Clinical strain (CIP, LVX, E, CC, TE, FOX) |
| 3. | *Staphylococcus aureus* KPD 740 | Clinical strain (E, CC, TE) |
| 4. | *Staphylococcus epidermidis* KPD 440 | Clinical strain (GM, CIP, LVX, E, CC) |
| 5. | *Staphylococcus hominis* KPD 910 | Clinical strain (TE, VA) |
| 6. | *Staphylococcus pettenkoferi* KPD 741 | Clinical strain (CIP, LVX, E, CC) |
| Gram-negative bacteria | | |
| 7. | *Acinetobacter baumannii* CRAB KPD 205 | Carbapenem-resistant clinical strain (PIP, TZP, CAZ, FEP, IMP, MEM, CIP, LVX, and SXT) |
| 8. | *Acinetobacter baumannii* MDR KPD 581 | Multidrug-resistant clinical strain (AMP, AMC, TZP, CEP, CXM, FOX, CTX, CAZ, FEP, ETP, MEM, CIP, SXT, and TOB) |
| 9. | *Acinetobacter baumannii* KPD 735 BA | Clinical strain (sensitive to all tested antibiotics) |
| 10. | *Enterobacter cloaceae*  KPD 297 | Clinical strain (AMP, AMC, TZP, CEP, CXM, FOX, CTX, CAZ, FEP, CSL, ETP, CIP, and SXT) |
| 11. | *Escherichia coli* KPD 217 | Clinical strain (AMP, AMC, TZP, CEP, CXM, FOX, CTX, CAZ, FEP, CSL, and SXT) |
| 12. | *Klebsiella pneumoniae* KPD 298 | Clinical strain (AMP, AMC, TZP, CEP, CXM, FOX, CTX, CAZ, FEP, ETP, IMP, MEM, AKN, CIP, SXT, and TOB) |
| 13. | *Pseudomonas aeruginosa* KPD 430 | Clinical strain (PIP, TZP, CAZ, FEP, CIP, LVX, TCC, and TOB) |
| 14. | *Pseudomonas aeruginosa* CRPA KPD 431 | Clinical strain (GM, PIP, TZP, CAZ, FEP, TCC, and MEM) |
| 15. | *Pseudomonas aeruginosa* KPD PAO 1 | (AMP, PIP, TZP) |

**^#^** Patterns of antibiotic resistance provided by the Department of Clinical Microbiology at University Clinical Centre, Gdansk, Poland, based on susceptibility tests performed according to the EUCAST for antibiotic resistance; PIP, Piperacillin; TZP, Piperacillin/tazobactam; CAZ, Ceftazidime; FEP, Cefepime; IMP, Imipenem; MEM, Meropenem; CIP, Ciprofloxacin; LVX, Levofloxacin; SXT, Trimethoprim/sulfamethoxazole; AMP, Ampicillin; AMC, Amoxicillin/clavulanic acid; AKN, amikacin; CEP, Cephalothin; CXM, Cefuroxime sodium; FOX, Cefoxitin; CTX, Cefotaxime; CSL, Cefoperazone/sulbactam; ETP, Ertapenem; TCC, Ticarcillin/clavulanic acid; TOB, Tobramycin; GM, Gentamicin.

## Supplementary Figures

**
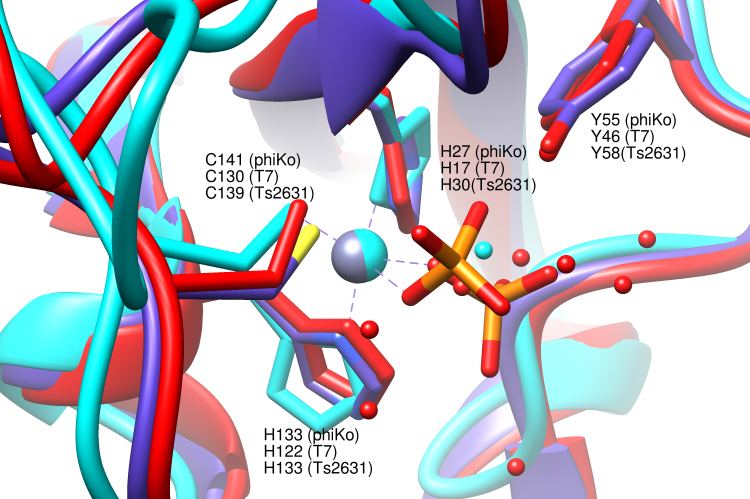
**


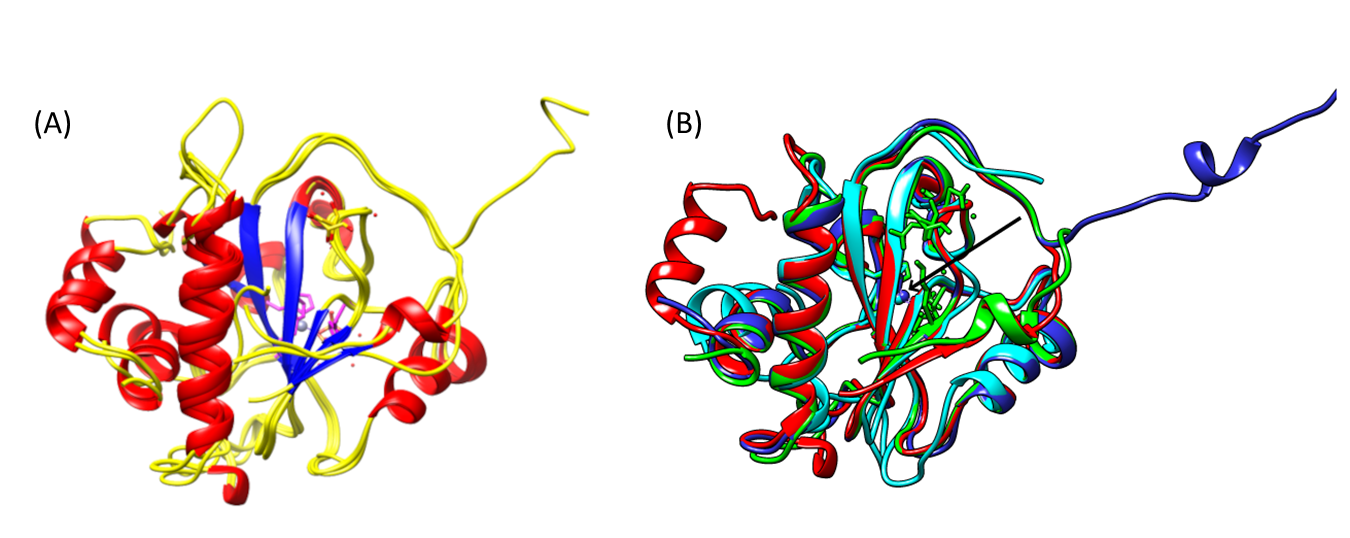
**Supplementary Figure 1.** The conservation of the catalytic center of the PhiKo enzyme (depicted in red), as compared to T7 lysozyme (cyan; PDB:1LBA) and Ts2631 endolysin (blue; PDB:6FHG). The 3D representation highlights the preservation of catalytic residues coordinating the Zn^2+^ ion across homologous proteins. Additionally, the structural conservation of the location of Tyr55 and its counterparts in T7 and Ts2631 is evident, emphasizing their essential role in phosphate (PO4) interaction.

**Supplementary Figure 2.** Structural modeling of the PhiKo enzyme: (A) Comparative structural alignment of models generated by various methods, including HHsearch+Modeller, Phyre2, OmegaFold, ColabFold (Alphafold2), and ESM2. The structures are color-coded by secondary elements: α-helices (red), β-sheets (blue), and loops (yellow) to highlight similarity of the models. (B) Structural alignment of PhiKo (red) with homologous proteins: T7 lysozyme (cyan; PDB: 1LBA; RMSD 0.91 for 98 residues), Ts2631 endolysin (blue; PDB: 6FHG; RMSD 0.71 for 130 residues), and ph2119 (green; PDB: 6SU5; RMSD 0.74 for 136 residues). Noteworthy differences in 3D superposition are primarily localized within loops, C-termini, and N-termini. C- and N- termini, while catalytic core is preserved (arrow).


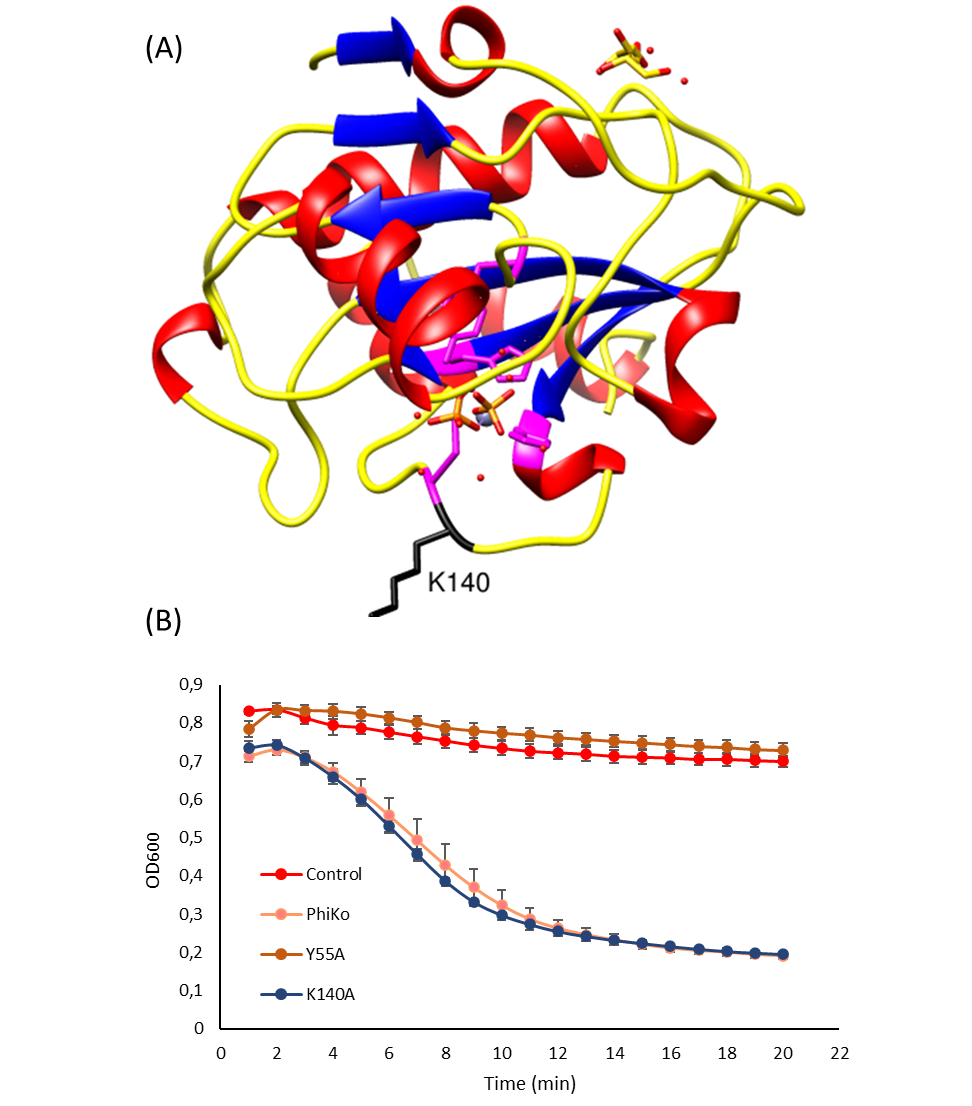


**Supplementary Figure 3.** The Lys140 is not essential for the PhiKo endolysin lytic activity. (A) Model of the PhiKo endolysin with Lys140 (K140) marked in black. The secondary-structure elements α-helices, β-strands, and loops are shown in red, blue, and yellow, respectively. (B) The results of the turbidity reduction assay show high lytic activity of the K140A mutant comparable the activity level of the wild-type protein. The Y55A mutant was inactive. Chloroform-treated *Thermus thermophilus* HB8 served as a substrate. Error bars represent means ± standard deviations. Experiment was repeated in triplicate.
